# Probing pathways by which rhynchophylline modifies sleep using spatial transcriptomics

**DOI:** 10.1186/s13062-023-00377-7

**Published:** 2023-05-05

**Authors:** Maria Neus Ballester Roig, Tanya Leduc, Julien Dufort-Gervais, Yousra Maghmoul, Olivier Tastet, Valérie Mongrain

**Affiliations:** 1 Department of Neuroscience, Université de Montréal, Montréal, QC H3T 1J4 Canada; 2 Center for Advanced Research in Sleep Medicine, Recherche CIUSSS-NIM, Montréal, QC H4J 1C5 Canada; 3 Department of Medicine, Université de Montréal, Montréal, QC H3T 1J4 Canada; 4 Centre de Recherche, Centre Hospitalier de l’Université de Montréal, 900 rue St-Denis, Tour Viger, Montréal, QC H2X 0A9 Canada

**Keywords:** Slow wave sleep, Sleep induction, Electrocorticographic oscillations, Molecular profiling, Hypothalamus, Sex

## Abstract

**Background:**

Rhynchophylline (RHY) is an alkaloid component of Uncaria, which are plants extensively used in traditional Asian medicines. Uncaria treatments increase sleep time and quality in humans, and RHY induces sleep in rats. However, like many traditional natural treatments, the mechanisms of action of RHY and Uncaria remain evasive. Moreover, it is unknown whether RHY modifies key brain oscillations during sleep. We thus aimed at defining the effects of RHY on sleep architecture and oscillations throughout a 24-h cycle, as well as identifying the underlying molecular mechanisms. Mice received systemic RHY injections at two times of the day (beginning and end of the light period), and vigilance states were studied by electrocorticographic recordings.

**Results:**

RHY enhanced slow wave sleep (SWS) after both injections, suppressed paradoxical sleep (PS) in the light but enhanced PS in the dark period. Furthermore, RHY modified brain oscillations during both wakefulness and SWS (including delta activity dynamics) in a time-dependent manner. Interestingly, most effects were larger in females. A brain spatial transcriptomic analysis showed that RHY modifies the expression of genes linked to cell movement, apoptosis/necrosis, and transcription/translation in a brain region-independent manner, and changes those linked to sleep regulation (e.g., *Hcrt*, *Pmch*) in a brain region-specific manner (e.g., in the hypothalamus).

**Conclusions:**

The findings provide support to the sleep-inducing effect of RHY, expose the relevance to shape wake/sleep oscillations, and highlight its effects on the transcriptome with a high spatial resolution. The exposed molecular mechanisms underlying the effect of a natural compound should benefit sleep- and brain-related medicine.

**Graphical Abstract:**

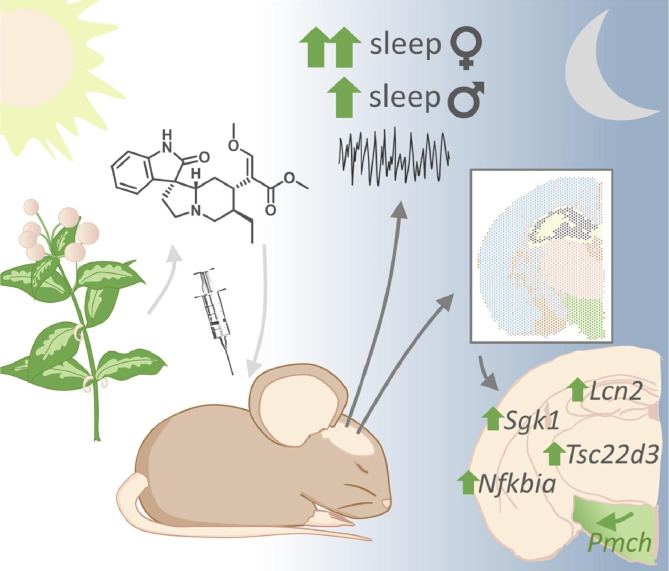

**Supplementary Information:**

The online version contains supplementary material available at 10.1186/s13062-023-00377-7.

## Background

Traditional Asian medicines have been used for centuries/millennia to alleviate psychiatric disease symptoms and ameliorate behaviors and states such as sleep [1]. Their natural origin is appealing for the general population, but the absence of solid empirical evidence and mechanisms of action has prevented their widespread use in the medical context [2]. Uncaria plants, in particular, were shown to have anticonvulsant, sedative and hypnotic effects, including positive impacts on psychiatric symptoms, sleep amount and quality in humans and mice [3–6]. Rhynchophylline (RHY) is an alkaloid component of Uncaria plants, and studies in rodents tend to support its hypnotic properties. For instance, RHY reduces locomotor activity, and was shown to increase sleep time when co-administered with pentobarbital in mice [7, 8]. Determining how RHY precisely affects wake/sleep states and their characteristic brain oscillations, and identifying the underlying mechanisms of action has definite value to support the use of Uncaria plants or RHY for sleep disturbances or other brain ailments.

Some studies have reported that RHY impacts neuronal firing in the rodent hippocampus and cerebral cortex [9–13], which suggests an effect on synchronized neuronal activity of the cerebral cortex occurring during wakefulness and sleep. Neuronal synchronization is generally reflected in the activity measured in slower frequencies of the electrocorticogram (ECoG); slow wave activity (SWA; 0.5–4.5 Hz) predominates during slow wave sleep (SWS), and theta activity (4–9 Hz) during paradoxical sleep (PS) [14–17]. Oscillatory activities not only represent defining features of sleep but also underly the role of sleep in brain function given, for instance, the established contributions of SWA during SWS and of theta activity during PS to memory consolidation [18–20]. Accordingly, it is crucial to determine the impact of sleep-inducing drugs on key oscillatory activities during sleep, and no such research exists for RHY.

RHY was shown to alter the cerebral cortex transcriptome in the APP/PS1 mouse model of Alzheimer’s disease, targeting molecular pathways related to the ubiquitin system, microglial function, and angiogenesis [21]. The effect of RHY on the genome-wide gene expression landscape of different brain regions in normal/healthy organisms remains to be established to adequately define the underlying mechanisms of its effect on the brain. Transcriptomic studies have proven particularly useful in the context of sleep research revealing gene expression signatures of wakefulness and sleep with, for instance, the mRNA level of genes linked to protein translation being increased by sleep, whereas that of immediate early genes associated to neurotransmission (e.g., *Fos*, *Arc*, *Homer1a*, *Egr1*), and of genes involved in stress responses (e.g., chaperones, heat shock proteins) being increased by sleep deprivation/extended wakefulness [22–30]. Moreover, the wake/sleep-dependent gene expression signature was shown to depend on the brain region [30–32]. Understanding effects of sleep-inducing compounds on the transcriptomic profile of brain regions controlling wake/sleep alternations and characteristic oscillations, such as the lateral hypothalamus (LH*)* [33–35], is required to understand the suitability of these compounds for sleep medicine.

We have here investigated how RHY modifies wake/sleep amount, alternation and ECoG oscillations, and interrogated underlying molecular mechanisms using a high spatial resolution transcriptomic approach. We uncovered that RHY increases the time spent in SWS at the expense of wakefulness, and changes the dynamics of key brain oscillations during SWS and wakefulness, with globally larger effects found in female in comparison to male mice. The spatial transcriptome revealed that RHY alters the expression of genes related to inflammation, apoptosis and the response to glucocorticoids; with some transcripts modified throughout the brain, and others for which changes were restricted to specific brain regions (e.g., genes linked to sleep regulation in the hypothalamus). This study demonstrates the relevance of traditional medicine for sleep disturbances, and of spatial transcriptomics to identify brain region-specific and sleep-relevant mechanisms.

## Results

### RHY increases SWS and state fragmentation

**Fig. 1:**
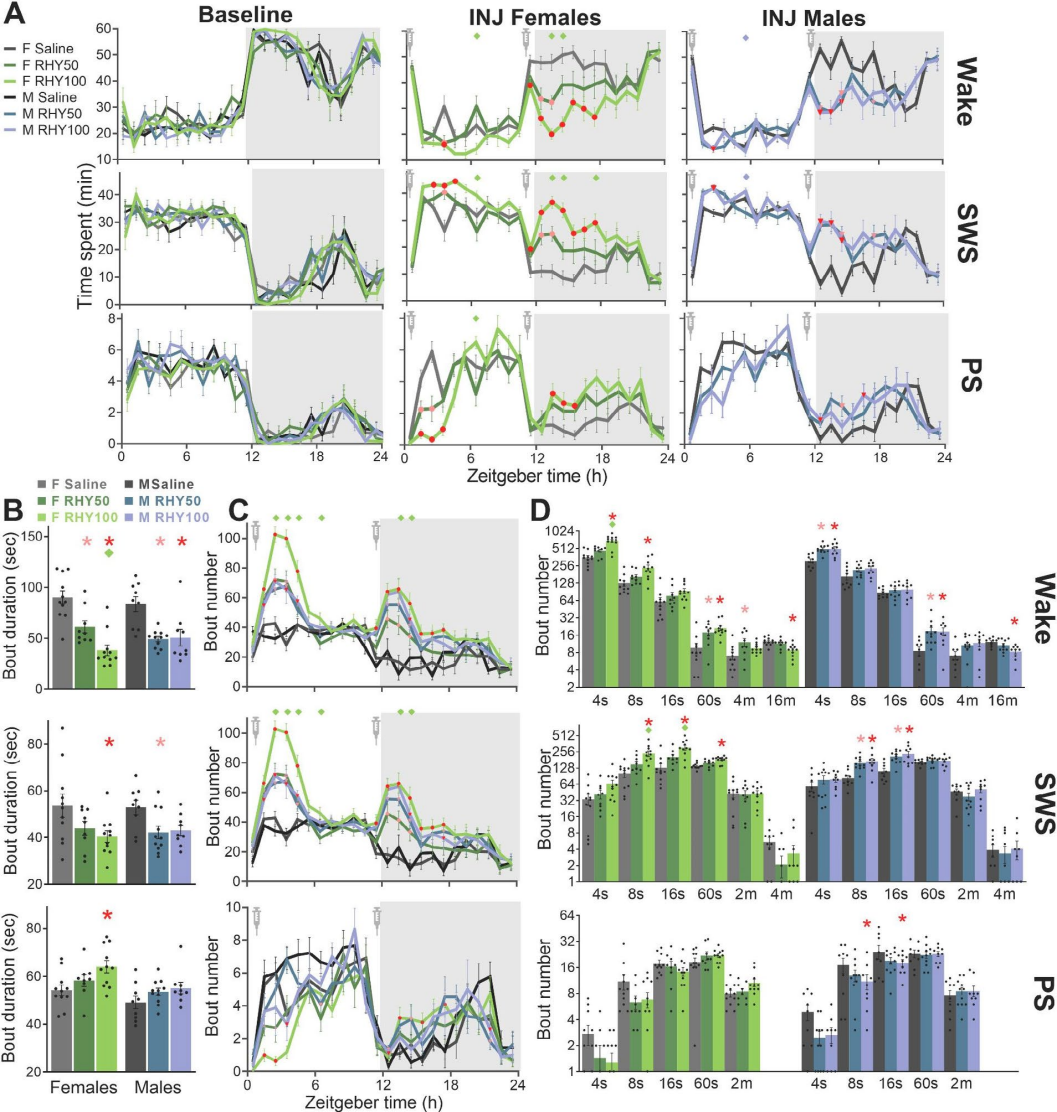
RHY increases SWS time, reduces wake time, and promotes wake and SWS fragmentation (**A**) Time course of minutes spent in each vigilance state (wake, SWS and PS) per hour during the baseline (BL) and injection (INJ) 24-h recordings. Significant interactions between RHY treatment and hour were found for wake, SWS and PS for both females (rANOVA: F_46,644_ > 2.0, p_adj_ < 0.01) and males (rANOVA: F_46,598_ > 1.9, p_adj_ < 0.05). Red and pink datapoints indicate significant differences compared to the saline group for each hour for the RHY100 and RHY50 groups, respectively (post hoc comparisons p < 0.05; same for panel C). Diamonds at the top of graphs indicate significant differences between RHY100 and RHY50 (green diamonds for females and blue diamonds for males, post hoc comparison p < 0.05; also in panels B to D). Grey backgrounds represent the dark period (also in C). (**B**) Mean duration of individual vigilance state bouts during the INJ day. Significant treatment effects were found for wake and SWS (females: F_2,28_ > 3.6, p < 0.05; males: F_2,26_ > 4.6, p < 0.02), and for PS in females (F_2,28_ = 4.4, p = 0.02). Red and pink stars indicate significant differences compared to the saline group of the same sex for RHY100 and RHY50, respectively (post hoc comparison p < 0.05; same for panel D). (**C**) Time course of the number of state bouts per hour for the INJ day. Significant interactions were found between RHY treatment and hour for all three states for females (rANOVA: F_46,644_ > 2.2, p_adj_ < 0.01) and males (rANOVA: F_46,598_ > 1.8, p_adj_ < 0.01). (**D**) Number of bouts of different durations for the INJ day. Significant treatment effects were found for specific duration during wake, SWS and PS (females: F_2,28_ > 3.7, p < 0.05; males: F_2,26_ > 3.8, p < 0.05).

Mice were submitted to ECoG recording before and after RHY treatment to investigate effects on vigilance states. Two doses of RHY were studied, 50 mg/kg (RHY50) and 100 mg/kg (RHY100), and compared to vehicle administration. Similar doses were previously reported to impact behavior, synaptic plasticity, and brain protein levels in mice [11]. Given the short (3–4 h) half-life/availability of RHY reported for the mouse and rat brain tissue [21, 36, 37], animals received two i.p. injection during the 24-h cycle, the first at light onset (Zeitgeber time 0: ZT0) and the second one hour before light offset (ZT11; with ZT12 representing light offset). This strategy also allowed to investigate time of day-dependent effects of RHY. Importantly, experiments were conducted in both female and male mice, and the effects of RHY was assessed in each sex to take into consideration the well-known sex differences in wake/sleep amount, alternation, and ECoG activity [38–40]. Sleep distribution and consolidation were equivalent between groups before RHY administration (Fig. 1A left column, and Fig. S1).

RHY was found to decrease time spent awake and increase time spent in SWS for 2 to 7 h after injection in both females and males (Fig. 1A). These effects were more prominent during the dark period (i.e., active period) for the two sexes (Fig. 1A and S1B), and larger in females than males when considering the early dark period (Fig. S1A). Interestingly, a dose-dependent effect was found for females, with RHY100 showing larger wake-suppressing and SWS-inducing effects than RHY50, while both doses impacted wake and SWS in a mostly similar manner in males (Fig. 1A and S1A). The effect of RHY on PS was highly dependent on time of day; with RHY reducing time spent in PS during the light period and increasing it during the dark period (Fig. 1A; in males, only significant for the dark period).

The mean duration and the number of individual bouts of wakefulness/sleep were interrogated to identify whether SWS was enhanced by prolonging individual bouts of SWS, by increasing the occurrence of SWS bouts or both. RHY was observed to significantly reduce the mean duration of wake and SWS bouts in both female and male mice (Fig. 1B). In parallel, RHY increased the number of wake and SWS bouts for 4 to 7 h after injection (Fig. 1C), with a highly similar time course for wakefulness and SWS. This effect was more prominent for shorter wake and SWS bouts (≤ 60 s; Fig. 1D) in comparison to longer SWS bouts (Fig. 1D and S1E), and also showed a dose-dependent effect for females in particular (Fig. 1B to D). Interestingly, RHY100 increased the duration of PS bouts only in females (Fig, 1B), and decreased the number of short PS bouts only in males (Fig. 1D). In sum, RHY shows wake-suppressing and SWS-inducing effects in mice, and impacts PS in a time of day-dependent manner. The SWS-inducing effect is driven by a higher occurrence of short individual bouts of SWS, resulting in an overall fragmentation of SWS. These effects are generally larger during the dark period and in females.

### RHY impacts the ECoG in a state-dependent manner

**Fig. 2:**
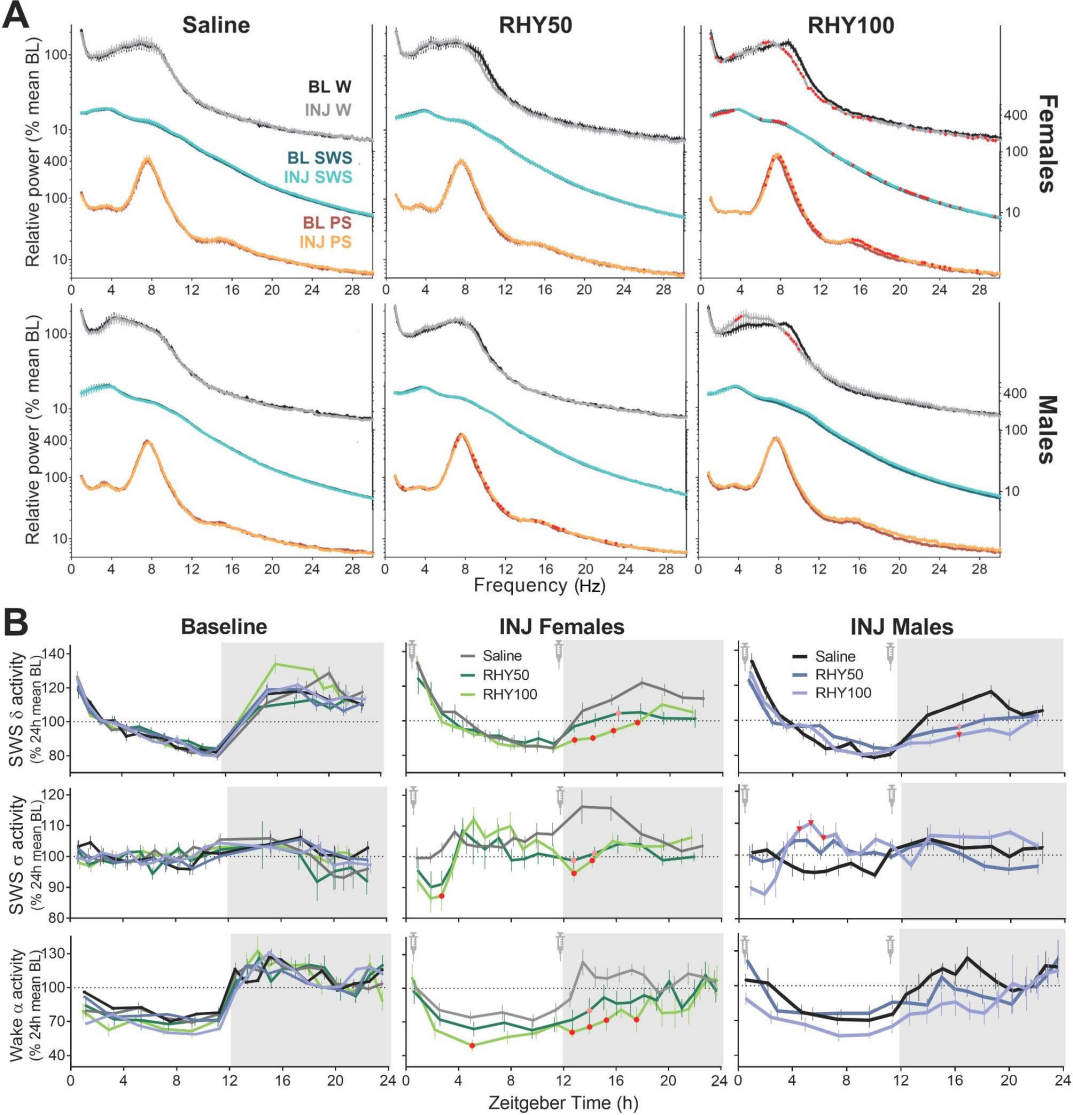
RHY modifies ECoG activity in a vigilance state-dependent manner (**A**) 24-h power spectra for each vigilance state during baseline (BL) and injection (INJ) recordings. Significant interactions between recording day (BL vs. INJ) and frequency bin were found for RHY100 wake (rANOVA females: F_116,928_ = 6.1, p_adj_ < 0.01; males: F_116,928_ = 4.8, p_adj_ < 0.05), for RHY100 SWS in females (F_116,928_ = 5.9, p_adj_ < 0.001), for RHY100 PS in females (F_116,928_ = 7.0, p_adj_ < 0.01) and for RHY50 PS in males (F_116,1044_ = 3.4, p_adj_ < 0.05). Red datapoints indicate significant differences between BL and INJ (planned comparisons p < 0.05). (**B**) 24-h time course of SWS delta activity (1–4 Hz; first row), SWS sigma activity (10–13 Hz; second row), and wake alpha activity (8–12 Hz; third row). For SWS, significant treatment by interval interactions were found for both sexes (delta: females F_34,442_ = 2.7, p_adj_ = 0.001; males F_34,391_ = 3.1, p_adj_ = 0.002; sigma: females F_34,442_ = 2.5, p_adj_ = 0.008; males F_34,391_ = 3.0, p_adj_ < 0.001). For wake alpha activity, a significant treatment by interval interaction was only found for females (females F_34,442_ = 1.6, p_adj_ = 0.03; males treatment by interval interaction F_34,357_ = 1.2, p_adj_ = 0.3 and main treatment effect F_2,21_ = 1.9, p = 0.2). Red and pink datapoints indicate significant differences compared to the saline group for each interval for the RHY100 and RHY50 groups, respectively (post hoc comparisons p < 0.05).

ECoG activity during wake, SWS or PS defines state quality and contributes to cognitive functioning, but has never been investigated after Uncaria or RHY treatments. Spectral analysis of the ECoG signal was here used to assess the impact of RHY on the 24-h power spectrum between 0.75 and 30 Hz of the three vigilance states. The ECoG spectral signature of wakefulness, SWS and PS was unaltered by saline in both females and males (Fig. 2A, first column). However, RHY100 was found to significantly affect the spectral composition of the ECoG during wakefulness in both females and males, and during SWS and PS in females (Fig. 2A, third column). In particular, the contribution of high theta/low alpha activity during wake was decreased by RHY100 (i.e., 8.0-12.25 Hz in females and 8.5–9.75 Hz in males) together with that in higher frequencies in females. In females, RHY100 also decreased the relative activity in delta frequencies (1–3 Hz) during SWS but generally increased activity in the theta/alpha (6.25–11.75 Hz) and beta (16–30 Hz) ranges during SWS and PS. RHY50 was also found to significantly impact PS power spectrum in males in a manner similar to that of RHY100 in females (Fig. 2A). Thus, RHY globally impacted the wake ECoG in frequencies generally linked to active wakefulness [41–43], as well as the sleep ECoG in frequencies contributing to cognitive processing [18–20].

The 24-h dynamics of ECoG activity in different frequency bands were then analyzed after RHY treatment. RHY significantly modified the dynamics of delta (1–4 Hz), theta (6–9 Hz) and sigma (10–13 Hz) activity during SWS (Fig. 2B and S1F). More precisely, SWS delta activity was decreased by RHY at a time matching with the strongest SWS-inducing effect (i.e., first half of the dark period). A dose-dependent effect was particularly prominent for females (and for faster delta; Fig. 2B and S1F). RHY impacted SWS theta and sigma activity in a similar manner: decreasing activity at the beginning of the light and dark periods in females, and increasing activity in the mid-light period in males (Fig. 2B and S1F). With regard to wakefulness, RHY modified the time course of alpha (8–12 Hz) activity by generally decreasing it throughout the 24-h cycle (only significant in females; Fig. 2B, bottom row). In sum, RHY alters ECoG activity during SWS and wakefulness in a manner that depends on time of day and sex.

### A specific RHY target correlates with sleep variables

**Fig. 3:**
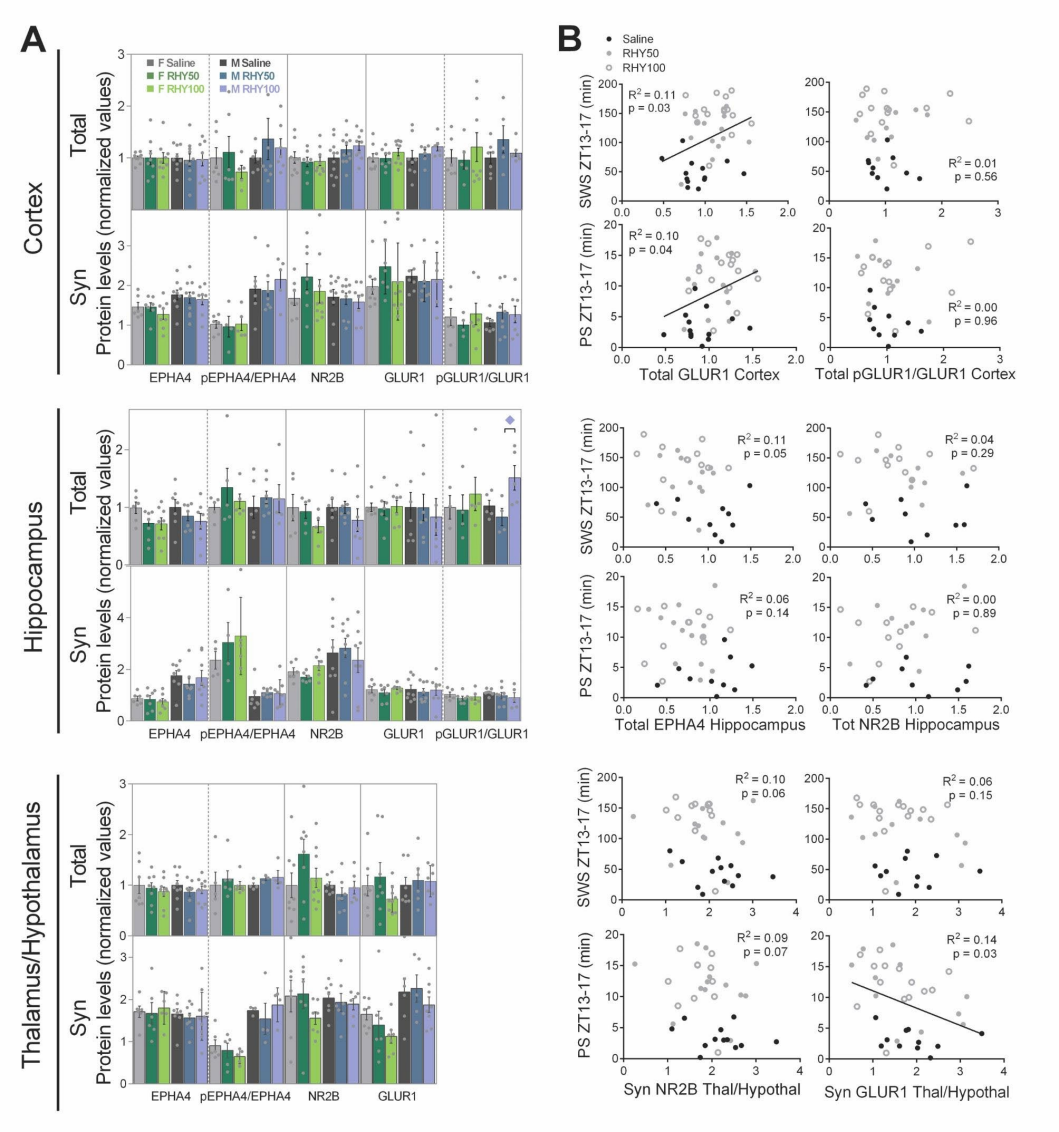
Target protein levels are generally not modified by RHY but correlated with sleep variables (**A**) Protein levels in total and synaptoneurosomal (Syn) fractions were quantified for the cerebral cortex, hippocampus, and a thalamus/hypothalamus spanning region. Except for the pGLUR1/GLUR1 ratio of the total protein fraction in the male hippocampus (F_2,11_ = 4.7, p = 0.03), no significant effect of treatment was found (see Fig. S2A for one way ANOVA results; n = 3–11 per group). The blue diamond at the top of a graph indicates a significant difference between RHY100 and RHY50 in males (post hoc comparison p < 0.05). (**B**) Correlations (including both female and male data) between protein levels and sleep variables showed significant correlations between cerebral cortex GLUR1 level (total fraction) and time spent in SWS or PS between ZT13 and ZT17; and between thalamic/hypothalamic GLUR1 (Syn fraction) and time spent in PS between ZT13 and ZT17.

To identify mechanisms underlying the widespread effects of RHY on wake/sleep quantity and quality, specific targets of RHY were investigated in the same mice submitted to ECoG recording, which were sacrificed 24 h after the first RHY injection. Total and synaptic protein levels were quantified for three brain regions selected for their contribution to the regulation of ECoG activity and/or sleep amount (i.e., cerebral cortex, hippocampus, and a region covering the thalamus and hypothalamus). Four targets, recently proposed as contributor of sleep-related effects of RHY [44], were examined (i.e., EPHA4, NR2B, GLUR1, and CDK5) together with GLT1, an astrocytic glutamate transporter regulated by EPHA4 [45, 46]. RHY did not significantly impact the protein level of these targets neither the phosphorylated level of EPHA4 in both total and synaptic fractions (Fig. 3A, S2A and S2B, and Table S1). However, the ratio between total phosphorylated GLUR1 (Ser845) and total GLUR1 was significantly increased by RHY100 in comparison to RHY50 in the hippocampus of male mice (Fig. 3A and S2A).

To tackle the large variability in protein measurements and simultaneously investigate relationships with specific sleep features, protein levels were correlated with sleep variables selected for their considerable/highest alteration by RHY (e.g., time spent in SWS between ZT13 and ZT17). Total GLUR1 level in the cerebral cortex was significantly associated with more time spent in SWS or PS at the beginning of the dark period, whereas synaptic GLUR1 in the thalamus/hypothalamus was found to be significantly associated with less time spent in PS at the same time (Fig. 3B). The ratio between phosphorylated GLUR1 and GLUR1 in total protein of the cerebral cortex was also negatively correlated with wake alpha activity measured in the first two-thirds of the dark period (Fig. S2C). In brief, even if the effect of RHY may not be captured by the targeted proteins when measured more than 12 h after the last RHY administration, GLUR1 could still contribute to alterations in specific sleep variables, including ECoG activity, under RHY treatment.

### RHY shapes the brain transcriptome in a time- and sex-dependent way

To precisely capture the mechanisms by which RHY impacts sleep, the experimental design was repeated in a second cohort of mice (not instrumented for ECoG recording) for which it was combined with a spatial transcriptomic approach. This was performed only with the higher dose of RHY, because of the stronger impact on sleep phenotypes. Mice were sacrificed 3 to 4 h after RHY100 (administered at ZT0 and ZT11) corresponding to the peak time of RHY effects (Fig. 1 and 2). Brains were thus sampled at ZT4 and ZT14 in females (F) and males (M) administered with saline (S) or RHY (R) (i.e., ZT4FS, ZT4FR, ZT14FS, ZT14FR, ZT4MS, ZT4MR, ZT14MS, ZT14MR). Coronal sections covering the cerebral cortex, hippocampus, and sleep-regulatory regions of the hypothalamus (e.g., LH) were processed using the 10x Genomics Visium Spatial Gene Expression kit coupled to RNA sequencing (RNAseq). The total number of reads and the mean number of reads per spatial spot under tissue were in the same order of magnitude between the eight different conditions (188 to 258 M, and 40 to 85 K, respectively), as well as the percent reads mapping to the genome (96.7 to 97.3 %).

**Fig. 4:**
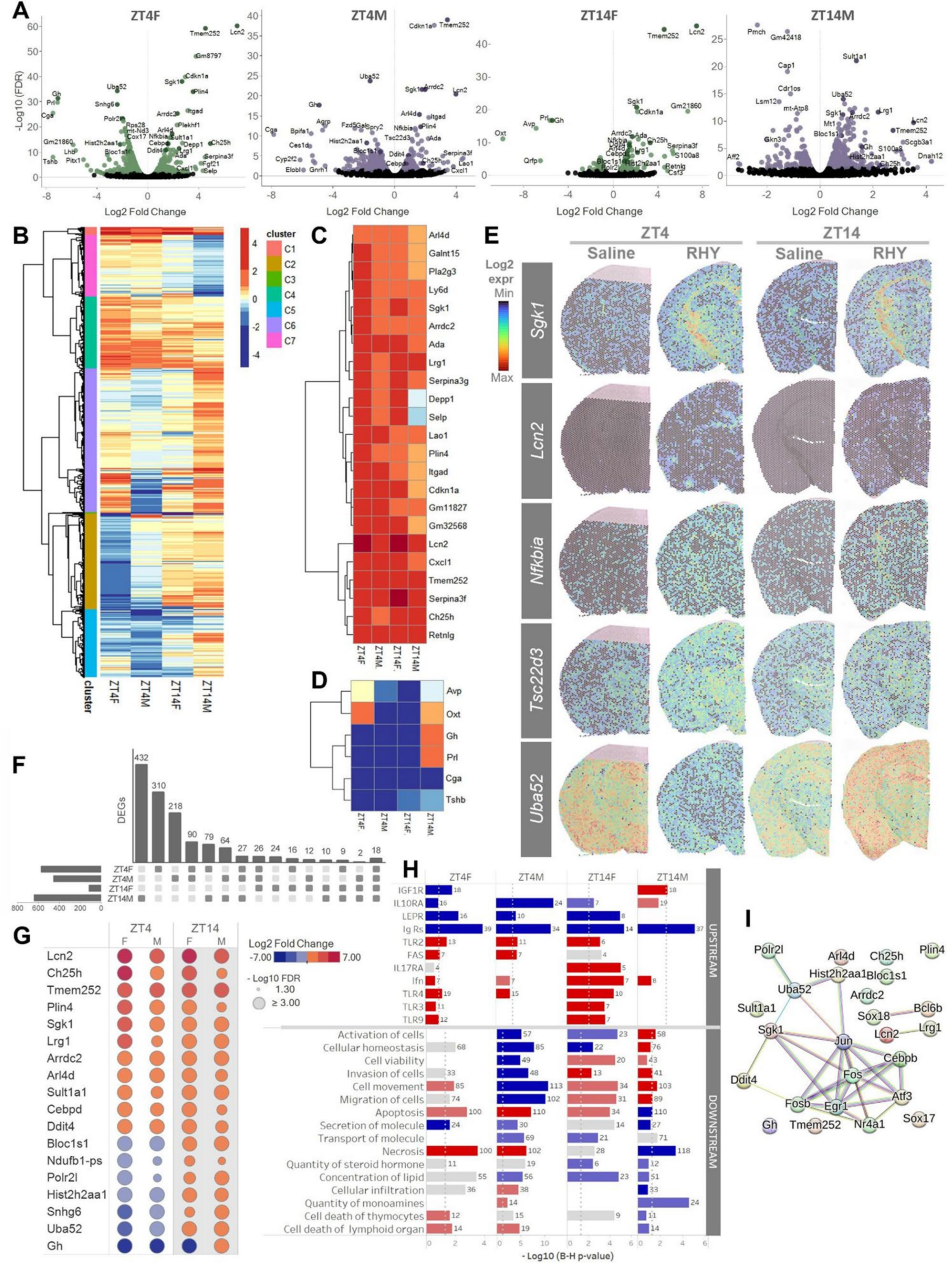
RHY modifies the mouse brain transcriptome (**A**) Volcano plots showing gene expression changes between RHY and saline conditions in female (F) and male (M) mice at ZT4 or ZT14. Green (female) and purple (males) datapoints indicate differentially expressed genes (DEGs; FDR < 0.05) between RHY100 and saline. Note that RHY changes gene expression in both directions. Black datapoints indicate transcripts without significant change. (**B**) Heatmap of the Log2 fold change in expression of the DEGs found for ZT4F, ZT4M, ZT14F, and ZT14M. The 1324 DEGs found in at least one of the four comparisons are shown. Automated hierarchical clustering showing seven DEG clusters (C1-C7) is also represented. (**C**) Zoom of cluster C1 showing 23 DEGs generally increased by RHY for ZT4F, ZT4M, ZT14F, and ZT14M. (**D**) Zoom of cluster C3 showing 6 DEGs generally decreased by RHY for ZT4F, ZT4M, ZT14F, and ZT14M. (**E**) Spatial maps of Log2 gene expression under saline and RHY treatments for female mice shown for *Sgk1*, *Lcn2*, *Nfkbia, Tsc22d3*, and *Uba52.* (**F**) Bar diagram illustrating the number of overlapping and non-overlapping DEGs among ZT4F, ZT4M, ZT14F, and ZT14M. (**G**) Table showing the fold change and significance level of the 18 DEGs common in all four comparisons. Hot colors indicate increases by RHY, and cold colors decreases. (**H**) Functional analysis of ZT4F, ZT4M, ZT14F and ZT14M DEGs showing enrichment of upstream receptors (top 11) and biological functions (bottom 16) extracted from Ingenuity pathway analysis (IPA). Hot colors indicate pathways predicted to be increased by RHY, and cold colors those decreased. Grey indicates no specific direction. The number of DEGs associated with each specific term is shown. Dotted grey lines indicate the significance threshold for enrichment (FDR < 0.05). (**I**) Representation of the protein-protein interaction network of the most enriched transcription factors (i.e., overlapping in 3 comparisons) made together with the 18 DEGs common in the ZT4F, ZT4M, ZT14F and ZT14M comparisons.

Transcriptome-wide gene expression data were first compared between RHY and saline for the full slice (i.e., all spatial spots, hereafter identified as bulk) for each time point and sex, resulting in four different sets of comparisons (i.e., ZT4F, ZT14F, ZT4M, ZT14M). This generated four sets of significant (i.e., Benjamini-Hochberg False Discovery Rate [FDR] < 0.05) differentially expressed genes (DEGs). RHY administration in the early light period resulted in 575 DEGs in females (ZT4F: 247 increased by RHY100, 328 decreased) and 457 DEGs in males (ZT4M: 126 increased, 331 decreased), and RHY administration in the evening generated 117 DEGs in females (ZT14F: 77 increased by RHY, 44 decreased), and 641 in males (ZT14M: 464 increased, 177 decreased; Fig. 4A and S3A, Table S2). A clustered heatmap compiling the fold change in expression of DEGs for ZT4F, ZT4M, ZT14F, and ZT14M identified seven distinct clusters (C1-C7; Fig. 4B and S3B). C1 and C4 comprised genes generally increased by RHY in the four comparisons (Fig. 4C and S3B); C3 featured six genes decreased by RHY in most comparisons (*Tshb*, *Cga*, *Prl*, *Oxt*, *Gh*, *Avp*; Fig. 4D and S3B); C2 and C7 genes generally increased or decreased by RHY in only one comparison or changed in opposite directions between ZT4 and ZT14; and C5 and C6 genes modified by RHY in different directions between ZT4 and ZT14 mainly for males (Fig. S3B). For instance, *Sgk1* and *Lcn2* belonging to cluster C1 were increased by RHY100 throughout the brain, with a particularly striking increase of *Sgk1* expression in white matter tracts (Fig. 4E). *Tsc22d3*, an example of C4 genes, was increased for ZT4F and ZT4M; while *Uba52* from C2 was decreased by RHY at ZT4 and increased at ZT14 (Fig. 4E).

Of importance is that even if C1, C3 and C4 comprised genes for which RHY impacted the expression in a generally consistent manner across the four different comparisons, the impact of RHY was highly dependent on time and sex. Indeed, the majority of DEGs were found in one single comparison (i.e., 310 for ZT4F, 218 for ZT4M, 24 for ZT14F, and 432 for ZT14M; Fig. 4F). An overlap of 90 DEGs was found between females and males at ZT4, whereas only 10 DEGs were common between sexes at ZT14 (Fig. 4F). In addition, fold changes in expression after RHY administration were significantly correlated between ZT4F and ZT4M samples and between ZT14F and ZT14M samples, whereas the correlations between time points within sex reached significance for females, but not for males (Fig. S3C). Interestingly, only 18 DEGs overlapped among the four comparisons, and the impact of RHY for seven of these was generally opposite at ZT4 in comparison to ZT14 (Fig. 4F-G).

### Gene ontology analysis of RHY time- and sex-dependent effects

The biological function analysis (Ingenuity Pathway Analysis: IPA) in ZT4F, ZT4M, ZT14F and ZT14M revealed that bulk DEGs were globally significantly enriched for genes linked to cell movement/migration, apoptosis, and necrosis (Fig. 4H, downstream). There was no overlap between all four DEG sets in gene enrichment for IPA canonical pathways, but some canonical pathways were significant in two out of the four comparisons (i.e., EIF2 signaling, oxidative phosphorylation, sirtuin signaling pathway, IL-17 signaling in fibroblasts, mitochondrial dysfunction, and glucocorticoid receptor signaling; Fig. S4A). Similarly, DAVID gene ontology analysis identified low overlap between comparisons, with ZT4F DEGs enriched in ribosome, mitochondrial and cell adhesion functions; ZT4M DEGs linked to transcription, extracellular region/cell adhesion, protein binding, hormone activity, apoptotic processes, cell development, and immune responses; ZT14F DEGs enriched in elements of the extracellular region, secretory granules and hormone activity; and ZT14M DEGs in extracellular region, mitochondria and ribosome function (Fig. S4D and Table S3). DEGs overlapping between ZT4F and ZT4M comparisons showed significant enrichment in transcriptional activation and PI3K/Akt signaling pathway, while overlapping DEGs between ZT14F and ZT14M were enriched in extracellular space and steroid metabolic process (Fig. S4E). In summary, functional analysis supports that the impact of RHY on the genome-wide gene expression signature of the targeted brain slice varies with time of day and sex.

### Core RHY-controlled genes are downstream of inflammation/immune pathways

Focusing on the 18 DEGs overlapping between the four comparisons in the bulk transcriptome can reveal core effects of RHY (i.e., independent of time of day, sex, and brain region). These 18 DEGs were related to glucocorticoid response (*Sgk1*, *Ddit4*, *Sult1a1*, *Ndufb1*, *PolR2L*), PI3K/Akt signaling (*Arld4*, *Ddit*4, *Gh*, *Sgk1*), lipid metabolism (*Cebpd*, *Ch25h*, *Lcn2*, *Plin4*), immune response (*Cebpd*, *Lcn2*, *Lrg1*), apoptosis (*Ddit4*, *Lcn2*, *Sgk1*), transcription (*PolR2L*, *Hist2h2aa1*, *Snhg6*), endosome processes (*Arrdc2*, *Bloc1s1*), oxidative phosphorylation (*Ndufb1-ps*), and ribosome assembly (*Uba52*) (Fig. 4G and S5). Interestingly, *PolR2L*, *Hist2h2aa1*, *Snhg6*, *Ndufb1-ps, Bloc1s1* and *Uba52* (linked to transcription, oxidative phosphorylation, or ribosome assembly) were decreased by RHY at ZT4, but increased at ZT14 (Fig. 4E, G and S5). A similar behavior was found for *Gh* in males.

A search for predicted upstream regulators of RHY-driven DEGs with IPA revealed that, globally, the four comparisons (i.e., ZT4F, ZT4M, ZT14F, and ZT14M) were enriched in genes downstream of specific transcription factors or their regulators (e.g., ESR1, NFKBIA, EGR1, SOX2, STAT1), immunoglobulin receptors, toll-like receptors (TLR) and cytokines (Fig. 4H, S4B, and S4C). This is strongly suggestive of an implication, among others, of cellular mechanisms related to the immune system. A second transcription factor enrichment analysis was conducted using CheA3 (Fig. S6A) [47]. Only two transcription factors were predicted as upstream regulators of RHY-driven transcriptomic changes by both IPA and CheA3: EGR1 and JUN (linked to the immune system and estrogen receptor signaling, respectively, and both to PI3K/Akt signaling). In fact, EGR1 and JUN are shown as central nodes in the protein-protein interaction network (String analysis) built from the transcription factor enrichment analysis with the 18 common DEGs (Fig. 4I). Overall, when considering the full mouse brain spatial landscape, RHY impacts the mRNA expression of genes downstream of inflammation/immune and estrogen receptor pathways.

### Brain region-specific effects of RHY on the transcriptome

**Fig. 5:**
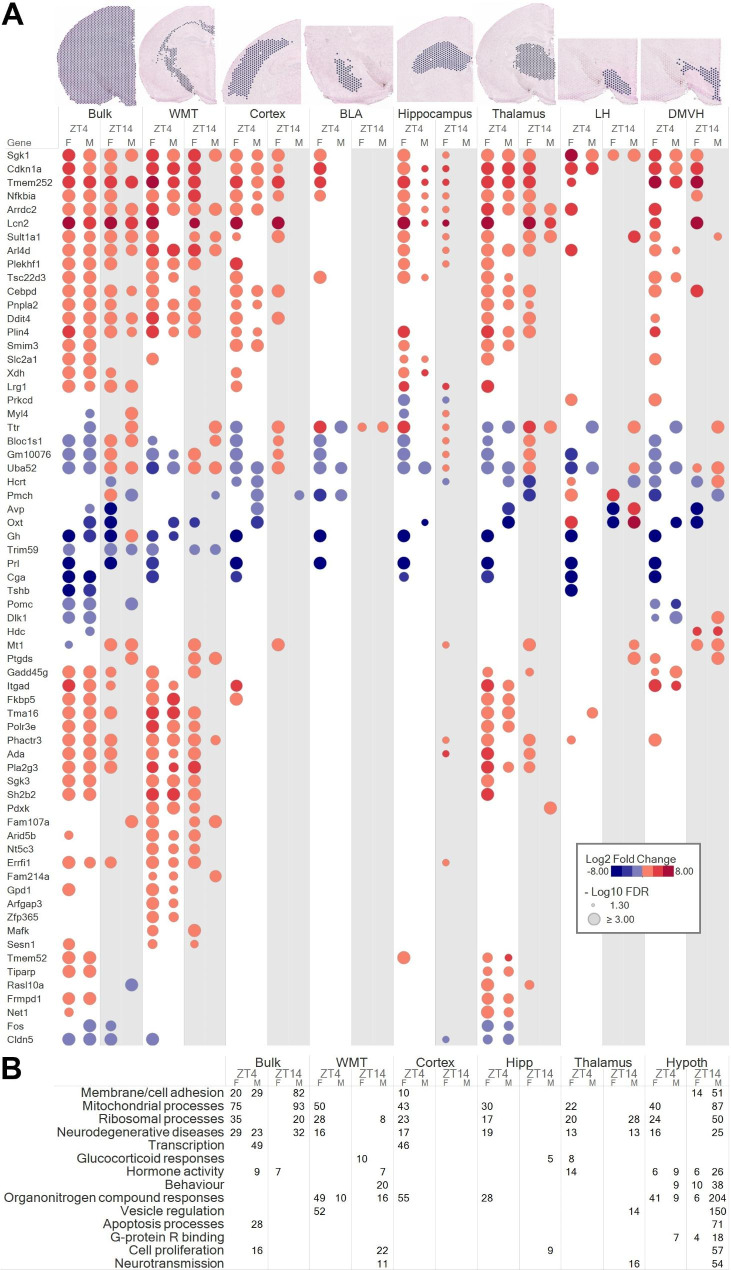
RHY modifies the brain transcriptome in a brain region-dependent manner (**A**) DEGs of regions of interest representing white matter tracts (WMT), cerebral cortex, basolateral amygdala (BLA), hippocampus, thalamus, lateral hypothalamus (LH) and dorsal, medial and ventromedial hypothalamus (DMVH). Bulk (full slice) data are also included for comparison. Only DEGs (FDR < 0.05) found in more than one comparison for at least one brain region are shown. Circle size indicates the significance level, hot colors indicate increase, and cold colors decrease. (**B**) Gene ontology enriched terms extracted from the DAVID annotation online tool for DEGs of the different brain regions. The number of DEGs enriched for the term category is indicated only for significant terms. The hypothalamus analysis includes LH and DMVH.

Importantly, the platform used for spatial transcriptomics allowed the identification of specific gene sets impacted by RHY in defined regions of the brain. The 10X Genomics automated clustering pipeline defined ensembles of spatial spots belonging to the same brain region, which was adequately represented by uniform manifold approximation and projection (UMAP) for dimension reduction (Fig. S6B). Subsequently, regions of interest of equivalent sizes and comprising spots corresponding to specific brain regions were defined in a semi-automated manner for saline and RHY brains (Fig. 5A). In the white matter capsules and tracts (WMT), 192 and 41 DEGs between RHY100 and saline were found at ZT4, and 59 and 108 at ZT14 (in females and males, respectively). In the cerebral cortex, 143 and 17 DEGs were found at ZT4, and 20 and 2 at ZT14 (females and males, respectively); in the basolateral amygdala (BLA), 115 and 3 at ZT4, and 1 and 4 at ZT14; in the hippocampus, 149 and 10 at ZT4, and 45 and 4 at ZT14; in the thalamus, 129 and 35 at ZT4, and 31 and 554 at ZT14; in the LH, 83 and 6 at ZT4, and 5 and 57 at ZT14; and finally in a region comprising dorsal, medial and ventromedial hypothalamus (DMVH), 138 and 24 at ZT4, and 17 and 132 at ZT14 (Table S2). This highlights that the impact of RHY is not restricted to a limited number of brain regions.

Some DEGs were common to most/many brain regions (e.g., *Sgk1*, *Cdkn1a*, *Tmem252*, *Nfkbia*, *Lcn2, Uba52*; Fig. 5A), and these were comprised in clusters C1, C4 or C7 of the previous bulk analysis. In contrast, many DEGs showed brain region specificity (Fig. 5A, Table S2). Indeed, some DEGs were found in only one region, such as *Myl4* in the hippocampus; *Pomc*, *Dlk1* and *Hdc* in DMVH; and many exclusive to WMT (*Aird5b*, *Nt5c3*, *Fam214a*, and *Gpd1*). The large number of DEGs in WMT may suggest an effect of RHY on axonal functioning. Assessing the effect of RHY in smaller regions (e.g., upper cortical layers, hippocampal pyramidal layers CA1, CA2, dentate gyrus granular layer, striatum, thalamic reticular nucleus) showed no DEG, which could result from the fewer number of spatial spots contributing to the comparisons. Not surprisingly, some region-dependent effects of RHY were different at ZT4 versus ZT14. For instance, hippocampal *Myl4* expression was decreased at ZT4 and increased at ZT14, and some thalamic DEGs were found only at ZT4 (e.g., *Tiparp*, *Frmpd1*, *Net1*, *Fos*).

DEGs found for the cerebral cortex, hippocampus, thalamus and hypothalamus (LH + DMVH) were enriched in biological/cellular/molecular functions generally in line with bulk DEGs (Fig. 5B, Table S3). These functions include cell adhesion, mitochondrial and ribosomal processes, as well as neurodegenerative diseases. Interestingly, enriched terms for the cerebral cortex and hippocampus were mainly linked to ZT4F DEGs, with much fewer significantly enriched terms found in males. An observation reminding the larger effects of RHY on wake/sleep variables in females in comparison to males (Fig. 1 and 2). DEGs found for the hypothalamus were also found to be enriched for hormone activity, behaviour and G-protein coupled receptor binding, functions less related to other brain regions in the present dataset. No significant enrichment for biological/cellular/molecular functions was found for the BLA. In sum, the transcriptomic analysis of different brain regions indicates that RHY alters gene expression with some level of region-specificity, and further supports an impact of time of day and sex. In addition, these findings suggest that RHY can modify wake/sleep architecture and ECoG activity by affecting the functioning of the cerebral cortex, hippocampus, thalamus and hypothalamus, together with that of main white matter tracts.

### RHY regulates sleep-related genes

**Fig. 6:**
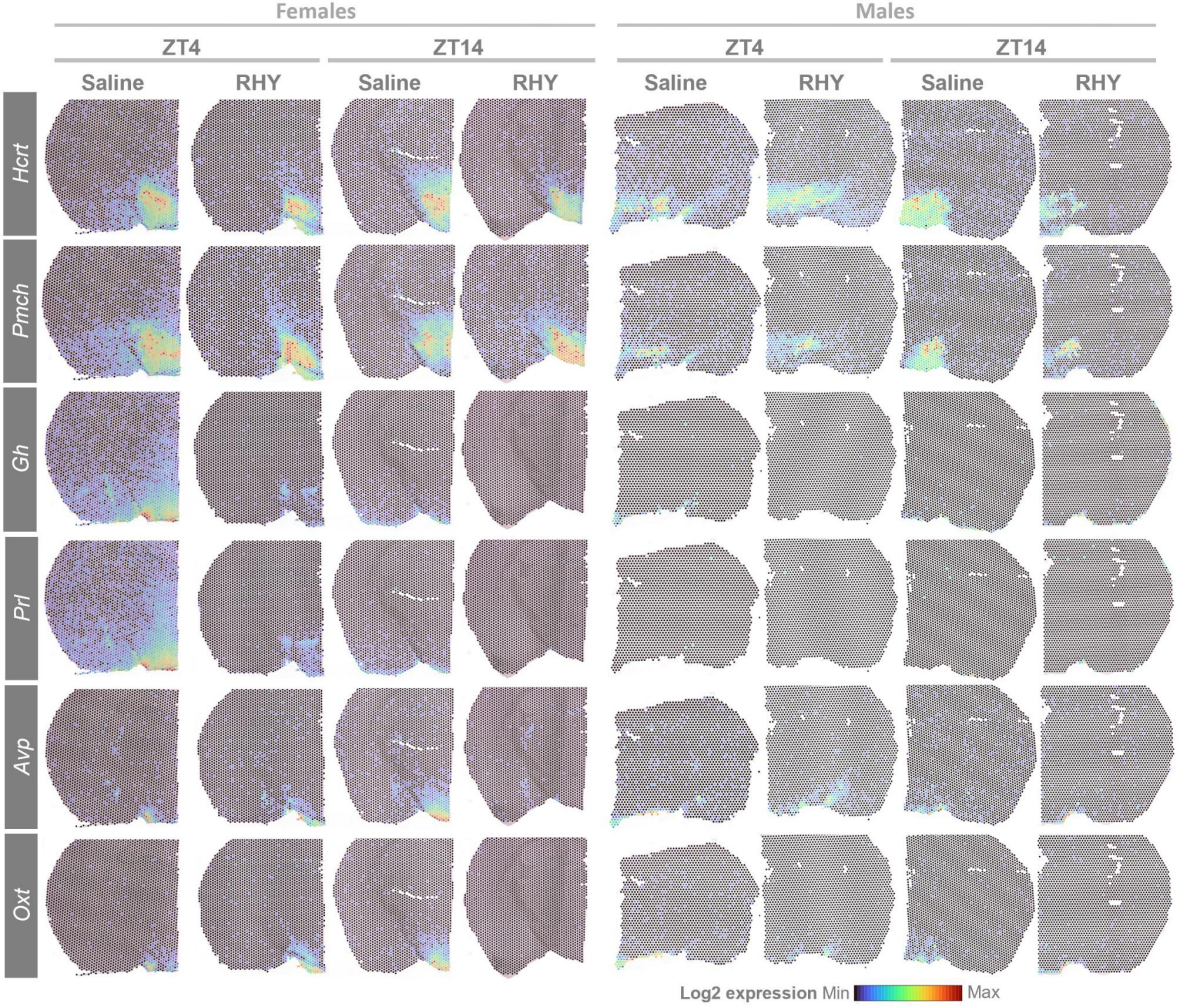
RHY modifies the expression of sleep-related genes Spatial map of Log2 gene expression at ZT4 and ZT14 under saline or RHY treatments in females (left four columns) and males (right four columns). Values were normalized separately for females and males. Warm colors indicate higher gene expression and cold colors lower gene expression.

Our genome- and brain-wide transcriptomic approach allowed for the identification of RHY-driven DEGs that are known regulators of wakefulness and sleep. First, RHY increased the expression of *Tsc22d3*, which codes for a protein responding to glucocorticoids and having anti-inflammatory roles, in most brain regions at ZT4 (Fig. 4E and 5A). *Tsc22d3* gene product is known as delta sleep-inducing peptide immunoreactor (DSIP) given its reported relationship with sleep amount [48, 49]. In addition, RHY decreased the expression of *Hcrt* in the cerebral cortex and thalamus, and modified it with different directions depending on the injection time in the hypothalamus (LH and DMVH; Fig. 5A and 6). RHY also generally decreased the expression of *Pmch* in several brain regions but increased it in the LH of female mice (Fig. 5A and 6). *Hcrt*- and *Pmch*-expressing neurons are well-recognized for their contributions in determining wake/sleep transitions and ECoG activity [33, 50, 51]. Of particular interest is that RHY appears to drive a spatial reorganization in the expression pattern of *Hcrt* and *Pmch* that was particularly marked in females in which RHY apparently restricts the spatial expression map to the LH at both ZT4 and ZT14 (Fig. 6).

RHY was also observed to alter the expression of several genes coding for peptides involved in the hypothalamic-pituitary axis (HPA). For instance, RHY100 generally decreased *Avp* in several studied brain regions (Fig. 5A and 6). Similar observations were made for *Oxt*, *Gh*, *Prl*, and *Cga*. *Tshb* and *Pomc* gene expression was decreased by RHY specifically in the hypothalamus and at ZT4 (Fig. 5A). *Pomc* codes for the precursor of several HPA peptides including corticotropin, melanocyte-stimulating hormone, and β-endorphin. Given the described bidirectional relationship between the HPA and sleep [52, 53], it is expected that these alterations participate in wake-suppressing and SWS-inducing effects of RHY. In addition, the HPA is under strong circadian control, and many of these HPA-related genes are expressed in a time of day-dependent manner (e.g., *Avp, Tshb, Pomc*) [54]. Therefore, the modulation of the circadian control of sleep by RHY is a likely route by which changes in wake/sleep amount and alternation can be generated.

## Discussion

We identified how a natural alkaloid component of Uncaria plants shapes wake/sleep amount, alternations, and ECoG activity, as well as multiple mechanisms contributing to these effects using a high spatial resolution transcriptomic strategy. Indeed, a sleep-inducing effect of RHY was exposed for mice in both females and males, which is in line with observations that drugs containing Uncaria increase sleep time in humans [3, 5, 6, 55]. An impact of RHY on wake/sleep ECoG activity was reported for the first time, indicative of a diversity of state-specific changes. We then revealed that RHY changes the transcriptomic landscape of the brain, affecting multiple brain regions in a manner that depends on time of injection and sex. Of importance is that RHY-driven modifications of the transcriptome comprised gene networks with key contributions to wake/sleep regulation in the hypothalamus, which represents a mechanism by which RHY can induce sleep.

The wake-suppressing and SWS-inducing effects of RHY, together with effects on ECoG activity, were found to depend on time of day. The larger effects found during the dark period in comparison to the light likely results from a ceiling effect related to the already high time spent in SWS during the light period in the healthy animals studied. In accordance with the well-described dynamics of homeostatic sleep pressure [56, 57], more SWS in the early dark phase is consistent with less delta activity during SWS at this same time of the day. Alternatively, the larger effect of RHY on time spent awake/asleep during the dark could emerge from an accumulative effect of the two consecutive doses of RHY. Our observations of RHY-driven changes in PS going in opposite directions between the light and dark periods, together with the short half-life of RHY in rodents [21, 36, 37], do not support this alternative explanation. Indeed, RHY decreased PS in the early light period but increased it in the dark period. It is possible that the initial suppression of PS created a PS pressure [58–60], leading to subsequent PS rebound after the second injection. It is particularly interesting that RHY decreased wake ECoG activity in a frequency range previously associated with the drive for homeostatic sleep need [43]. This overall indicates that RHY shapes wake/sleep quality in parallel to wake/sleep amount, which needs to be carefully considered in sleep medicine.

Mechanisms underlying the impact of RHY on wake/sleep were first investigated at the level of selected individual targets previously related to sleep [44]. In brains sampled 13 h after the last RHY injection, no change in NR2B, EPHA4, pEPHA4, GLUR1, GLT1, or CDK5 were found. The lack of effect on NR2B, pEPHA4 and GLUR1 contrasts with previous observations made using similar and longer delays between RHY administration and brain sampling [61–63]. However, these previous studies reported effects in rodent disease models only (e.g., epilepsy, depression, addiction) and used different administration paradigm. It is thus possible that more subtle effects in healthy animals were not captured by the current study design, especially given findings of significant relationships between levels of GLUR1 (and pGLUR1/GLUR1) and selected sleep variables measured about 10 h before. We thus aimed at better defining the mechanisms underlying RHY-driven wake/sleep alterations by precisely focusing on the time delays with highest effects and applying a comprehensive spatial interrogation.

RHY was found to have widespread effects on the brain transcriptome, affecting hundreds of transcripts throughout the brain, generally associated to apoptosis, necrosis, and cell movement/migration. In addition, the core 18 genes modified by RHY independent of time of injection and sex were linked to pathways and cellular functions previously described to be impacted by RHY (e.g., PI3K/Akt signaling, immune response/inflammation) [21, 44, 64–66]. Interestingly, there is a considerable overlap between the present RHY-driven DEGs and genes reported to be modulated by the wake/sleep history [22, 23, 26–28, 30, 67–74]. For instance, total sleep deprivation (SD) was found to increase the expression of *Sgk1*, *Gh*, *Nfkbia*, and *Cdkn1a* in the cerebral cortex and hippocampus [69, 72], while PS restriction increased *Lcn2* but reduced *Pmch* and *Oxt* expression [68, 71]. *Tsc22d3* also represents a target commonly impacted by RHY and SD [26, 27, 30, 72–74], which has a particular relevance to sleep regulation as indicated before. Accordingly, RHY alters biological functions known to respond to sleep loss (e.g., stress responses, apoptotic processes, cytokine and TNF signaling, transcriptional processes, cell differentiation) [22, 23, 27, 28, 30, 67, 70, 73]. This finding that RHY impacts gene networks responding to SD reveals a route by which it can promote sleep. We hypothesize that RHY is inducing a specific molecular program encompassing multiple brain regions and favoring sleep. Importantly, RHY-driven gene expression changes were dependent on the timing of brain sampling, which is fully in line with the well-know effect of daytime/circadian time on the brain transcriptome [29, 54]. This suggests that the molecular reprograming induced by RHY differs between the early light and early dark periods as an adaptation to the specific internal transcriptional state of the brain.

A main discovery of the current study concerns the impact of RHY on hypothalamic regions. In the hypothalamus, RHY affected genes related to hormone activity and behaviour, which likely contributes to behavioral observations reported here and by other studies [7, 8, 61, 75, 76]. RHY was notably found to modify the expression of *Hcrt* and *Pmch* in the LH and DMVH. HCRT neurons project to cortical and thalamic areas, and activation of these cells was found to induce behavioral arousal and neuronal arousal/desynchronization of the cortex [33, 77, 78]. MCH neurons were shown to fire during sleep [79], and the activation of these cells induces PS [50, 51], or both SWS and PS in the dark phase [80]. Thus, the impact of RHY on hypothalamic *Hcrt* and *Pmch* expression pattern could represent a key mechanism by which RHY modulates time spent in wakefulness/sleep. In females in particular, the spatial reorganization of hypothalamic *Pmch* expression and its increase in the LH might directly contribute to wake-suppression/SWS-induction.

Sex differences in sleep amount and synchronized cortical activity are well-known in humans and rodents [39, 81–85]. Here, we have highlighted that many effects of RHY are different in female mice in comparison to males, which was particularly striking with regard to wake/sleep architecture, ECoG activity, and spatial transcriptome. It is interesting to note that dose-dependent effects on wake/sleep amount and ECoG were only found for females, and thus that males would not benefit from a higher dosage in the tested range. The generally larger effects of RHY on sleep variables in females was paralleled by a generally stronger impact on the transcriptome, particularly during the light period. Sex differences in sleep are often attributed to gonadal hormones [40, 86], but sex differences in absorption, metabolism, or downstream effectors [87–90] of RHY are also potential contributors to the generally higher effects seen in females. Our findings convincingly support the need to systematically consider both females and males when investigating the potential of a drug for sleep disturbances.

## Conclusions

This study depicts RHY as an alkaloid herb derivative with great promise for sleep medicine. This was done using a detailed identification of its effects on wakefulness and sleep states at the level of architecture and synchronized cortical activity, combined to an exhaustive examination of the brain transcriptome. We have exposed potential key sleep-relevant mechanisms of action of RHY in the brain, which include gene networks with roles in the immune system/inflammation and hormone signaling, and contributions of several brain regions comprising the hypothalamus. Given that the transcriptome does not always reflect changes occurring at protein and functional levels [91, 92], future research questioning the translatome, proteome, and phosphoproteome will be needed to further increase the understanding of the way by which RHY induces sleep. Furthermore, the present study has investigated a single coronal brain slice selected to represent a variety of regions with relevance to sleep, but follow-up investigations should consider other important sleep-relevant regions such as the ventrolateral preoptic hypothalamus and the brainstem to fully decipher how RHY benefits sleep.

## Methods

### Animals, surgeries, and protocols

Male and female C57BL/6J were bred on site. Animals were housed in a 12 h light / 12 h dark cycle, at 24 ± 1 °C, with water and food available *ad libitum*. All protocols were approved by the *Comité d’éthique de l’expérimentation animale* of the *CIUSSS-NIM*. For wake/sleep characterization, female (n = 31 [n = 11 Saline, n = 9 RHY50, n = 11 RHY100], 12.7 ± 2.0 w old, 20.7 ± 1.7 g; all virgin) and male (n = 29 [n = 9 Saline, n = 11 RHY50, n = 9 RHY100], 12.0 ± 3.2 w old, 26.3 ± 1.7 g; all virgin) mice were implanted with ECoG and electromyography (EMG) electrodes under deep Ketamine/Xylazine anaesthesia (120/10 mg/kg, i.p. injection) as detailed previously [93, 94]. Following recovery, mice were habituated to cabling conditions for one week before experiments. ECoG/EMG signals were then recorded during 48 h comprising 24 h of undisturbed/baseline (BL) conditions and 24 h under injection (INJ) conditions (consisting in two intraperitoneal injections, one at ZT0 [i.e., light onset] and one at ZT11 [i.e., 1 h before light offset] of saline, RHY50 or RHY100). Mice were sacrificed between ZT0 and ZT1 immediately after the INJ day, and brain regions were immediately dissected and frozen on dry ice. For spatial transcriptomics, saline and RHY100 treatments were compared. Two females and two males received saline (one of each sex at ZT0 and one of each sex at ZT0 and ZT11; all virgin), and two females and two males received RHY100 (one of each sex at ZT0 and one of each sex at ZT0 and ZT11; all virgin). Mice were sacrificed at ZT4 or ZT14, and brains were immediately sampled, frozen on dry ice together with embedding in OCT compound.

### RHY preparation

Two doses of RHY were tested and compared with vehicle (saline: NaCl 0.9 %): RHY 50 mg/kg (RHY50) and RHY 100 mg/kg (RHY100). RHY (Baoji Herbest Bio-Tech Co., Ltd, # 76-66-4) was diluted in NaCl 0.9 %, and homogenized the day before administration. Dilutions were kept at 4 °C until use. The detailed methods can be found in Supplementary Material.

### Protein extraction and immunoblotting

Brain regions were processed to extract total and synaptoneurosomal (SYN) proteins similar to previously performed [93, 95]. Fifteen µg of protein were loaded on gels, separated by SDS-PAGE, and transferred to PVDF membrane. Membranes were blocked, incubated with primary antibodies, and then with secondary antibodies as detailed in supplementary methods. Membranes were revealed using Odyssey CLx imaging system (LI-COR). Additional information can be found in Supplementary Material.

### Tissue and library preparation for spatial transcriptomics

Tissue preparation was conducted according to the 10x Genomics Visium Spatial Gene Expression protocol (CG000240 Rev C). Ten µm coronal slices were cut at -23 °C with a cryostat (HM525 NX Thermo Scientific or CM3050S Leica), and slices at ∼1.5 mm posterior to the bregma were mounted on chilled Visium Spatial Gene Expression slides (10x Genomics). Slides were then incubated in a thermocycler, and immersed in Methanol. For Hematoxylin-Eosin staining (10X Genomics protocol CG000160 Rev A), slides were covered with isopropanol and, after air dry, covered with Hematoxylin. After washing, slides were covered with Bluing Buffer, and covered with Eosin mix. Lastly, slides were dried, and imaged using an Axio Imager M2 microscope (Zeiss, Canada). Libraries were prepared according to 10x Genomics Visium Spatial Gene Expression protocol (CG000239 Rev D). Briefly, immediately after imaging, brain slices were covered with permeabilization enzyme, covered with a reverse transcription master mix, and incubated at 53 °C for 45 min. The resulting cDNA was incubated with 0.08 M KOH, washed with EB buffer, and incubated with second strand synthesis mix. Slices were denatured, and samples were mixed with cDNA amplification mix for amplification (98 °C for 3 min; 15 cycles of 15 s at 98 °C, 20 s at 63 °C and 1 min at 72 °C; 1 min at 72 °C). cDNA was cleaned with SPRIselect beads (Beckman Coulter, Cat# B23318), and submitted to cDNA fragmentation, end repair and A-tailing. Post-ligation cleanup was done again with SPRIselect. Sample indexes i5 and i7, and amplification mix were added to the samples for amplification (45 s at 98 °C; 15 cycles of 98 °C for 20 s, 67 °C for 30 s, 72 °C for 20 s; and 72 °C for 1 min). Then, cDNA was purified with SPRIselect, and libraries were stored at -20 °C.

### ECoG analyses

The ECoG was analyzed per 4-s epochs similar to previously described [93, 94]. Total time spent in each vigilance state, and the mean duration of individual bouts of vigilance states were averaged for the 12 h light and dark periods. The total number of bouts of different durations was calculated for the 24 h BL or INJ, for each state separately. Hourly time-courses were calculated for mean time spent in each state and the total number of bouts. The bipolar ECoG signal was submitted to spectral analysis conducted using a Fast Fourier transform (FFT). Activity was calculated for the full 24 h between 0.75 and 30 Hz with a 0.25 Hz resolution. Power spectra of the 24-h INJ recording were expressed as a percent of the mean power of all 0.25-Hz bins of all states during the 24-h BL for each mouse. The time course of defined frequency bands was calculated using averages per time interval as done previously [94, 96]. Vigilance state variables were compared using one-way analyses of variance (ANOVAs), two-way ANOVAs, and two-way repeated-measure ANOVAs (rANOVA) (with Greenhouse-Geisser or Huynh-Feldt correction if appropriate). Significant interactions were decomposed using planned comparisons or post hoc Tukey tests. Data are reported as mean and standard error of the mean (SEM), and the threshold for statistical significance was set to 0.05.

### Protein level quantification and statistical analyses

Bands from immunoblots were analyzed using ImageJ (NIH) [97]. Band intensity was quantified by calculating the area under the curve for the averaged pixel intensity along the vertical plane, which was normalized to actin, to a control sample (included on all membranes), and to the average of the total protein of the saline treatment. Values normalized to actin and control sample for phosphorylated and non phosphorylated forms of EPHA4 and GLUR1 were used to calculate the phosphorylation ratio, and were then normalized to the average ratio of the saline treatment. Protein levels were compared using one-way ANOVAs, and decomposed using post hoc Tukey tests. Pearson correlations were computed between protein levels and sleep variables measured during time of higher effect of RHY (i.e., between ZT13 and ZT17).

### RNA sequencing and analyses

Paired-end dual indexed RNAseq was conducted using an Illumina NovaSeq6000 SP100 sequencer (Genome Quebec Innovation Centre, Montreal, Canada), at a sequencing depth of approximately 150 M read pairs per sample (> 40 K reads per spot under tissue). RNAseq was performed according to instructions of 10x Genomics for the Visium Spatial Gene Expression kit: read 1, 28 cycles; i7 index read, 10 cycles; i5 index read, 10 cycles; read 2, 90 cycles. Sequencing reads were aligned to the reference mouse genome (mm10) using the Space Ranger “spaceranger count” pipeline. Then, the pipeline “spaceranger aggr” was used to find genes differentially expressed between ZT4S and ZT4R samples, and between ZT14S and ZT14R samples for females and males. Gene-spot matrices were analysed using Loupe Browser, and DEGs were considered significant when FDR < 0.05 (Benjamini-Hochberg correction for multiple comparisons) [98]. Common DEGs between time point and sex were analyzed using VIB/UGent Bioinformatics & Evolutionary Genomics Venn diagram online calculator and R version 4.1.2. Clustered heatmap was created using the Ward.D2 clustering method. “Spaceranger aggr” was run again using ZT4S, ZT4R, ZT14S, and ZT14R to obtain figures of spatial gene expression normalized per slide. DEG lists were introduced in the DAVID annotation online tool, the Kyoto Encyclopedia of Genes and Genomes (KEGG) pathway annotation online tool, and the Ingenuity Pathway Analysis software (IPA, Qiagen) for functional analyses. Transcription factor enrichment analysis was performed with the online tool ChIP-X Enrichment Analysis Version 3 (ChEA3) [47], and subsequent analysis of functional protein association networks was done with the online database STRING [99].

## Electronic supplementary material

Below is the link to the electronic supplementary material.

Supplementary Material 1

Supplementary Material 2

Supplementary Material 3

Supplementary Material 4

## Data Availability

RNA-sequencing data have been deposited in the GEO database (https://www.ncbi.nlm.nih.gov/geo), and will be publicly available on the date of publication under accession numbers GSE217058 and GSE218537.

## List of abbreviations

ANOVA: Analysis of variance
BLA: Basolateral amygdala
ChEA3: ChIP-X Enrichment Analysis Version 3
DEG: Differentially expressed gene
DMVH: Dorsal, medial and ventromedial hypothalamus
ECoG: Electrocorticogram
EMG: Electromyography
FFT: Fast Fourier transform
INJ: Injection
IPA: Ingenuity Pathway Analysis
KEGG: Kyoto Encyclopedia of Genes and Genomes
LH: Lateral hypothalamus
PS: Paradoxical sleep
RHY: Rhynchophylline
RNAseq: RNA sequencing
SD: Sleep deprivation
SEM: Standard error of the mean
SWA: Slow wave activity
SWS: Slow wave sleep
SYN: Synaptoneurosomal
UMAP: Uniform manifold approximation and projection
WMT: White matter capsules and tracts
ZT: Zeitgeber time
