# Supplementary material for: Probing pathways by which rhynchophylline modifies sleep using spatial transcriptomics
Source: Biol Direct. 2023 May 5;18:21. doi: 10.1186/s13062-023-00377-7 (PMC10161643; doi:10.1186/s13062-023-00377-7)
Supplement: Supplementary file 1 — Supplementary Material 1 [file 13062_2023_377_MOESM1_ESM.pdf]

Supplementary figures and methods

Probing pathways by which rhynchophylline modifies sleep using spatial transcriptomics

Maria Neus Ballester Roig, Tanya Leduc, Julien Dufort-Gervais, Yousra Maghmoul, Olivier Tastet, Valérie Mongrain

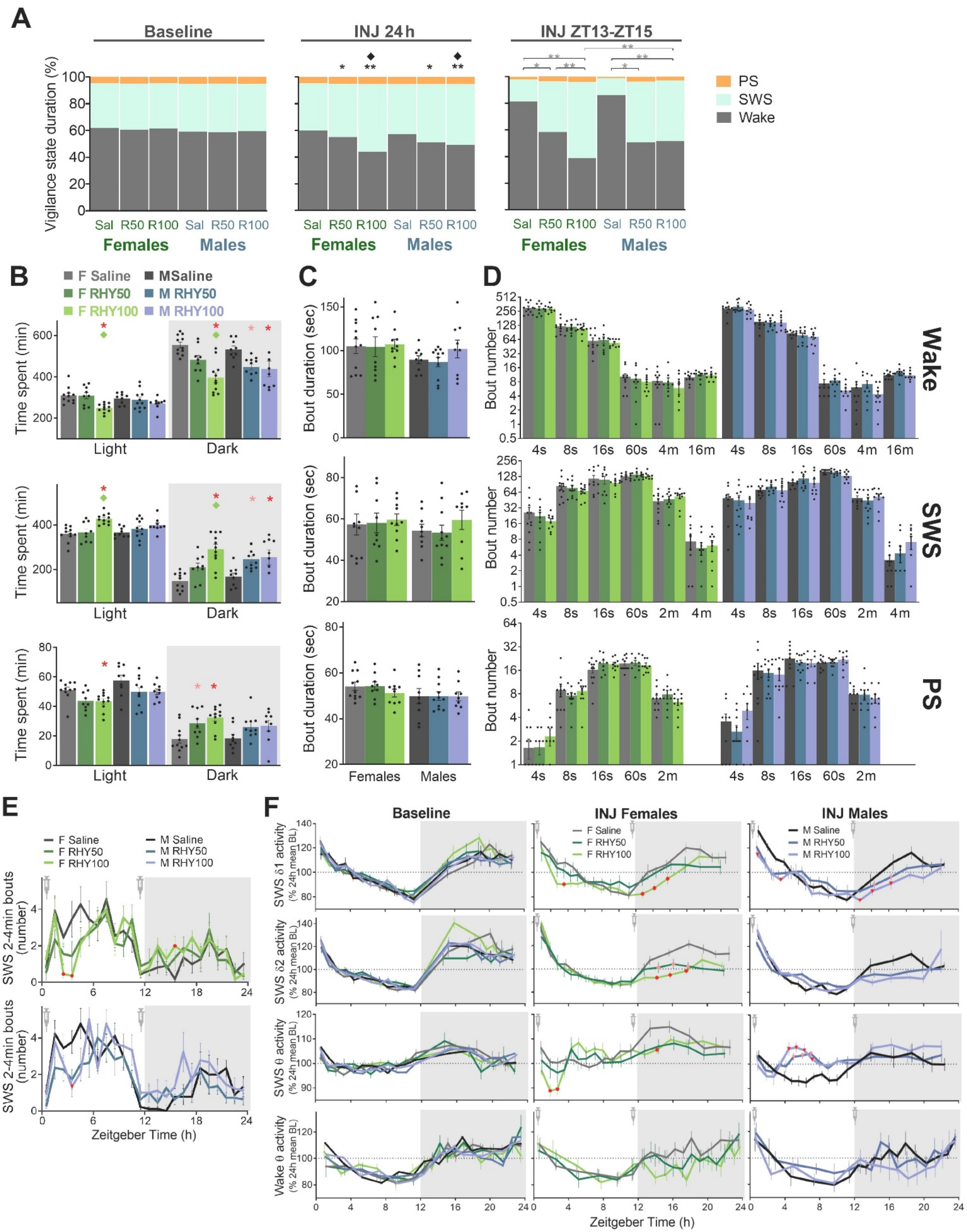

Figure S1. Groups were similar in baseline, RHY increases SWS more prominently in females, and modifies 24-h dynamics of ECoG oscillations in a vigilance state-dependent manner.

(A) Percent time spent in each vigilance state for the 24-h baseline or injection (INJ) recordings (first and second panels, respectively), and for the ZT13-ZT15 interval during the INJ recording (right panel). For the 24-h INJ, significant treatment effects were found for wake and SWS ($F_{2,54} > 19.2$, $p < 0.001$; stars indicate significant differences from saline and diamonds from RHY50: post hoc comparisons *$p < 0.05$, **$p < 0.01$). For the ZT13-ZT15 interval, a significant treatment by sex interaction was found for wake ($F_{2,54} > 3.2$, $p < 0.05$; stars indicate significant differences between indicated points: post hoc comparisons *$p < 0.05$, **$p < 0.01$).

(B) Total time spent in wake, SWS and PS calculated separately for the light and dark periods during the 24-h INJ recording. For females, RHY significantly changed wake, SWS and PS for both the light and dark periods ($F_{2,28} > 4.7$, $p < 0.01$). For males, RHY significantly affected wake and SWS only for the dark period ($F_{2,26} > 4.4$, $p < 0.05$). Red and pink stars indicate significant differences from the saline group for RHY100 and RHY50, respectively, and diamonds differences from RHY50 (post hoc comparisons $p < 0.05$). Grey backgrounds represent the dark period (also in E and F).

(C) Mean duration of individual bouts of wake, SWS and PS during the baseline day.

(D) Number of bouts of different duration for wake, SWS and PS during the baseline day.

(E) Number of longer bouts (2-4 min) of SWS for the INJ day. Significant treatment by hour interactions were found (rANOVA: females $F_{46,644} = 1.5$, $p_{adj} = 0.03$; males $F_{46,598} = 1.8$ $p_{adj} = 0.004$). Red datapoints indicate significant differences between the saline and RHY100 groups (post hoc comparisons $p < 0.05$).

(F) Baseline and INJ 24-h dynamics of spectral activity in different frequency bands during SWS and wakefulness. During INJ day, treatment by interval interactions were found for SWS δ1 activity (delta 1: 0.75-2 Hz; females $F_{34,442} = 2.5$, $p_{adj} < 0.001$; males: $F_{34,374} = 2.6$, $p_{adj} < 0.001$), for SWS δ2 activity (delta 2: 2.5-4 Hz; females $F_{34,442} = 2.6$, $p_{adj} < 0.01$; males: $F_{34,374} = 3.4$, $p_{adj} < 0.001$), for SWS θ activity (theta: 6-9 Hz; females $F_{34,442} = 3.0$, $p_{adj} < 0.001$; males: $F_{34,374} = 2.8$, $p_{adj} < 0.001$). No interaction was found for baseline and for wake θ activity during INJ day. Red and pink datapoints indicate significant differences compared to the saline group for each interval for the RHY100 and RHY50 groups, respectively (post hoc comparisons $p < 0.05$).

A	CORTEX			
	FEMALES		MALES	
EPHA4 (TOT)	$F_{2,23} = 0.00$	$p = 1.00$	$F_{2,24} = 0.04$	$p = 0.96$
EPHA4 (SYN)	$F_{2,18} = 0.86$	$p = 0.44$	$F_{2,24} = 0.19$	$p = 0.83$
pEPHA4 (TOT)	$F_{2,15} = 1.66$	$p = 0.22$	$F_{2,18} = 0.24$	$p = 0.79$
pEPHA4 (SYN)	$F_{2,16} = 1.40$	$p = 0.28$	$F_{2,16} = 1.29$	$p = 0.30$
pEPHA4/EPHA4 (TOT)	$F_{2,14} = 0.67$	$p = 0.53$	$F_{2,18} = 0.40$	$p = 0.68$
pEPHA4/EPHA4 (SYN)	$F_{2,12} = 0.05$	$p = 0.96$	$F_{2,15} = 0.29$	$p = 0.75$
NR2B (TOT)	$F_{2,21} = 0.22$	$p = 0.81$	$F_{2,28} = 1.58$	$p = 0.22$
NR2B (SYN)	$F_{2,20} = 0.91$	$p = 0.42$	$F_{2,24} = 0.12$	$p = 0.88$
GLUR1(TOT)	$F_{2,23} = 0.82$	$p = 0.45$	$F_{2,17} = 0.92$	$p = 0.42$
GLUR1(SYN)	$F_{2,21} = 0.86$	$p = 0.44$	$F_{2,18} = 0.11$	$p = 0.90$
pGLUR1(TOT)	$F_{2,18} = 0.43$	$p = 0.66$	$F_{2,28} = 2.43$	$p = 0.11$
pGLUR1(SYN)	$F_{2,16} = 0.29$	$p = 0.75$	$F_{2,24} = 0.10$	$p = 0.90$
pGLUR1/GLUR1(TOT)	$F_{2,16} = 0.34$	$p = 0.72$	$F_{2,16} = 1.42$	$p = 0.27$
pGLUR1/GLUR1(SYN)	$F_{2,14} = 0.29$	$p = 0.75$	$F_{2,18} = 0.46$	$p = 0.64$
CDK5 (TOT)	$F_{2,23} = 1.57$	$p = 0.23$	$F_{2,24} = 1.44$	$p = 0.26$
CDK5 (SYN)	$F_{2,23} = 2.11$	$p = 0.14$	$F_{2,24} = 0.16$	$p = 0.86$
GLT1(TOT)	$F_{2,22} = 0.04$	$p = 0.96$	$F_{2,16} = 0.85$	$p = 0.45$
GLT1(SYN)	$F_{2,18} = 0.65$	$p = 0.54$	$F_{2,18} = 0.11$	$p = 0.90$
	HIPPOCAMPUS			
	FEMALES		MALES	
EPHA4 (TOT)	$F_{2,19} = 2.30$	$p = 0.13$	$F_{2,18} = 1.02$	$p = 0.38$
EPHA4 (SYN)	$F_{2,17} = 0.25$	$p = 0.78$	$F_{2,21} = 0.51$	$p = 0.61$
pEPHA4 (TOT)	$F_{2,14} = 0.50$	$p = 0.61$	$F_{2,17} = 0.29$	$p = 0.75$
pEPHA4 (SYN)	$F_{2,14} = 0.48$	$p = 0.63$	$F_{2,21} = 0.22$	$p = 0.80$
pEPHA4/EPHA4 (TOT)	$F_{2,12} = 0.69$	$p = 0.52$	$F_{2,15} = 0.28$	$p = 0.76$
pEPHA4/EPHA4 (SYN)	$F_{2,10} = 0.58$	$p = 0.58$	$F_{2,20} = 0.27$	$p = 0.77$
NR2B (TOT)	$F_{2,12} = 1.13$	$p = 0.35$	$F_{2,18} = 0.70$	$p = 0.51$
NR2B (SYN)	$F_{2,15} = 2.32$	$p = 0.13$	$F_{2,21} = 0.27$	$p = 0.77$
GLUR1(TOT)	$F_{2,17} = 0.03$	$p = 0.97$	$F_{2,18} = 1.12$	$p = 0.89$
GLUR1(SYN)	$F_{2,16} = 0.77$	$p = 0.48$	$F_{2,21} = 0.12$	$p = 0.89$
pGLUR1(TOT)	$F_{2,10} = 0.53$	$p = 0.61$	$F_{2,11} = 0.48$	$p = 0.63$
pGLUR1(SYN)	$F_{2,14} = 0.62$	$p = 0.55$	$F_{2,18} = 3.15$	$p = 0.07$
pGLUR1/GLUR1(TOT)	$F_{2,9} = 0.32$	$p = 0.73$	**$F_{2,11} = 4.73$**	**$p = 0.03$**
pGLUR1/GLUR1(SYN)	$F_{2,14} = 0.53$	$p = 0.60$	$F_{2,17} = 0.69$	$p = 0.52$
CDK5 (TOT)	$F_{2,19} = 0.90$	$p = 0.42$	$F_{2,12} = 0.03$	$p = 0.97$
CDK5 (SYN)	$F_{2,15} = 1.38$	$p = 0.28$	$F_{2,21} = 0.44$	$p = 0.65$
GLT1(TOT)	$F_{2,19} = 0.20$	$p = 0.82$	$F_{2,18} = 0.88$	$p = 0.43$
GLT1(SYN)	$F_{2,16} = 1.13$	$p = 0.35$	$F_{2,20} = 0.70$	$p = 0.51$
	THALAMUS/HYPOTHALAMUS			
	FEMALES		MALES	
EPHA4 (TOT)	$F_{2,21} = 0.28$	$p = 0.75$	$F_{2,24} = 0.60$	$p = 0.56$
EPHA4 (SYN)	$F_{2,15} = 0.12$	$p = 0.88$	$F_{2,23} = 0.09$	$p = 0.91$
pEPHA4 (TOT)	$F_{2,15} = 0.42$	$p = 0.73$	$F_{2,9} = 0.66$	$p = 0.54$
pEPHA4 (SYN)	$F_{2,15} = 1.29$	$p = 0.31$	$F_{2,10} = 0.09$	$p = 0.92$
pEPHA4/EPHA4 (TOT)	$F_{2,14} = 0.19$	$p = 0.83$	$F_{2,8} = 0.91$	$p = 0.44$
pEPHA4/EPHA4 (SYN)	$F_{2,13} = 0.90$	$p = 0.43$	$F_{2,8} = 0.41$	$p = 0.68$
NR2B (TOT)	$F_{2,22} = 1.70$	$p = 0.21$	$F_{2,17} = 0.75$	$p = 0.49$
NR2B (SYN)	$F_{2,18} = 1.07$	$p = 0.36$	$F_{2,17} = 0.18$	$p = 0.83$
GLUR1(TOT)	$F_{2,21} = 1.27$	$p = 0.30$	$F_{2,17} = 0.11$	$p = 0.90$
GLUR1(SYN)	$F_{2,18} = 1.37$	$p = 0.28$	$F_{2,16} = 0.61$	$p = 0.56$
pGLUR1(SYN)			$F_{2,15} = 1.08$	$p = 0.36$
CDK5 (TOT)	$F_{2,19} = 0.22$	$p = 0.81$	$F_{2,24} = 1.40$	$p = 0.27$
CDK5 (SYN)	$F_{2,17} = 0.04$	$p = 0.96$	$F_{2,23} = 0.60$	$p = 0.56$
GLT1(TOT)	$F_{2,21} = 0.79$	$p = 0.47$	$F_{2,18} = 0.06$	$p = 0.94$
GLT1(SYN)	$F_{2,18} = 1.86$	$p = 0.18$	$F_{2,17} = 0.01$	$p = 0.99$

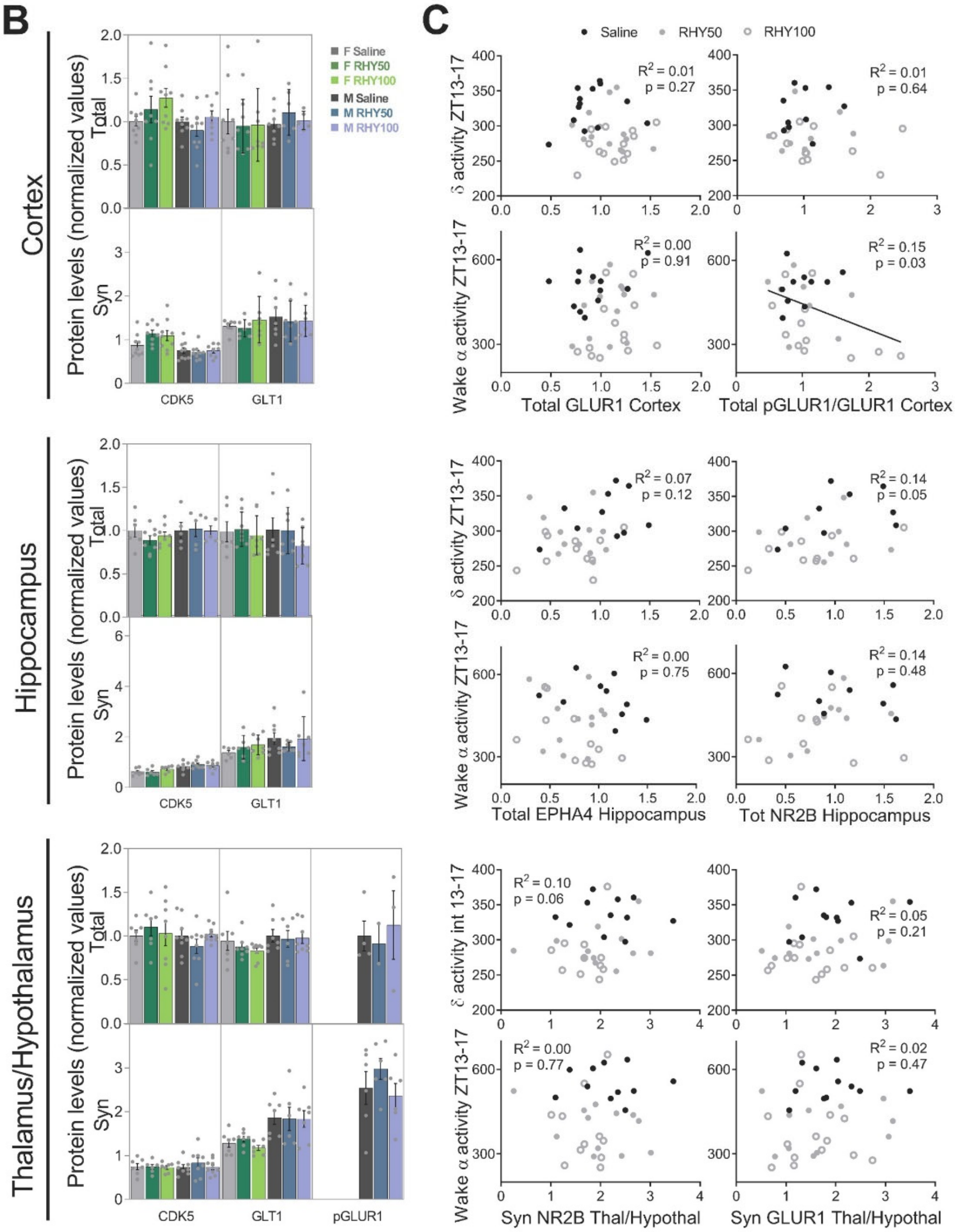

Figure S2. CDK5 and GLT1 levels were unchanged by RHY and pGLUR1 correlates with decreased wake alpha activity

(A) Results of one-way ANOVAs comparing protein levels in total (Tot) and synaptoneurosomal (Syn) fractions between treatments (Saline, RHY50 and RHY100) computed separately for female and male mice.

(B) Quantifications of CDK5 or GLT1 in the total and synaptoneurosomal (Syn) protein fractions of the cerebral cortex, hippocampus and thalamus/hypothalamus region (one-way ANOVAs in S2A).

(C) Correlations (including both female and male data) between protein levels and SWS delta (δ) activity and wake alpha (α) activity averaged between ZT13 and ZT17. The ratio of phosphorylated GLUR1 to GLUR1 in the cerebral cortex (total fraction) was negatively correlated with wake alpha activity.

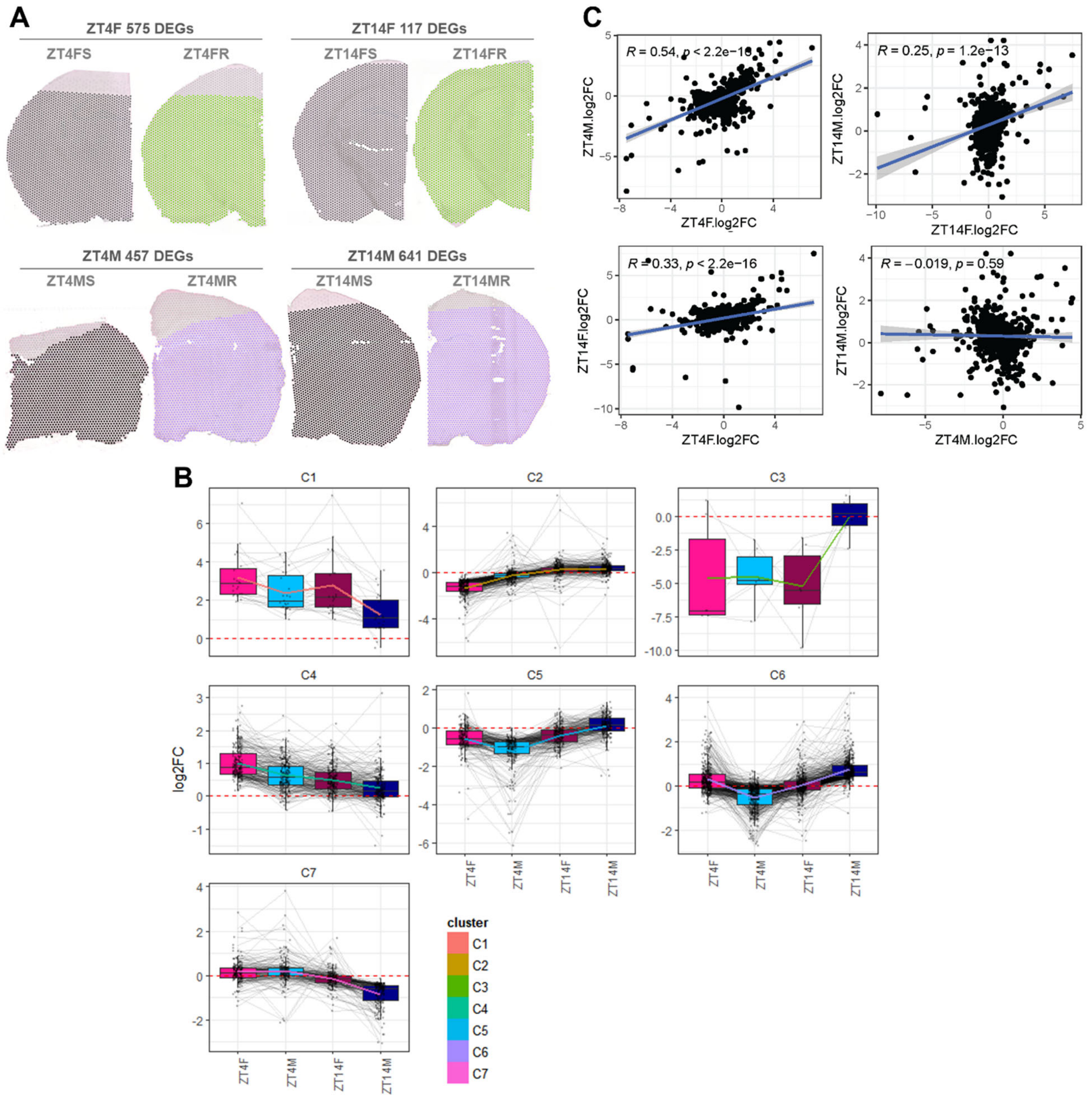

Figure S3. Analysis of differentially expressed genes (DEGs) and hierarchical DEG clusters.

(A) Spatial spots used to calculate differentially expressed genes (DEGs) between the different RHY and saline conditions**.** Common spots between slices were used to conduct comparisons. These common spots are shown in green for females and purple for males for the RHY100 conditions and in dark blue for the saline conditions. The number of DEGs obtained for each comparison is indicated at the top of each pair (FDR < 0.05).

(B) Box plots showing the behavior of genes belonging to seven clusters (C1-C7) obtained from automated clustering of Log2 fold change of DEGs from the four comparisons between RHY100 and saline conditions (ZT4F, ZT4M, ZT14F and ZT14M).

(C) Correlations of changes in gene expression between sexes or between time points. Positive correlations were found for the Log2fold change of DEGs modified by RHY at the same time of injection in different sexes (upper panels), and for RHY effects on female samples taken at different time-points (lower left panel), but no correlation was found between ZT4M and ZT14M (lower right panel).

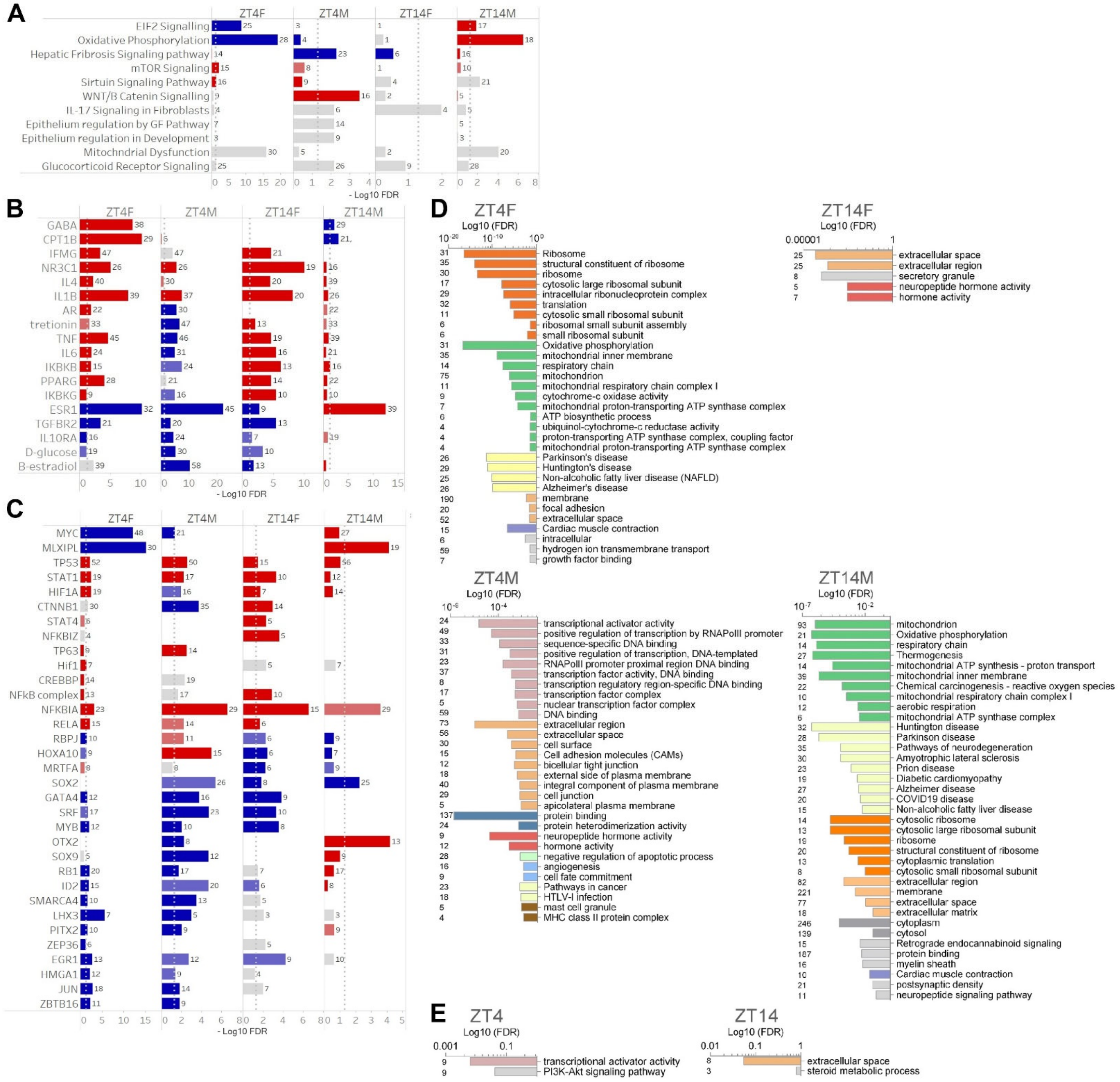

Figure S4. Enrichment in canonical pathways, potential upstream regulators, and functional gene ontology terms

(A-C) Analysis of enrichment using Ingenuity pathway analysis (IPA). DEGs of each four comparisons (ZT4F, ZT4M, ZT14F, ZT14M) were used to assess their potential enrichment for canonical pathways (A), and for upstream regulators, including upstream elements (B) and transcription factors (C). The bar size indicates enrichment significance (-Log10 FDR), and the bar color indicates if terms are predicted to be activated (hot colors), inhibited (cold colors), or enriched with no particular direction (grey). The number of genes found for each term is shown. Dotted lines indicate the threshold for statistical significance.

(D-E) DAVID database gene ontology terms for functional annotation found enriched for the DEGs (FDR < 0.05) in each dataset (D) and in the list of DEGs common in the two ZT4 comparison datasets (ZT4 DEGs) or in the two ZT14 comparison datasets (ZT14 DEGs) (E). Enriched terms are colored by related functions and organized by FDR value. The number of DEGs related to each term is indicated.

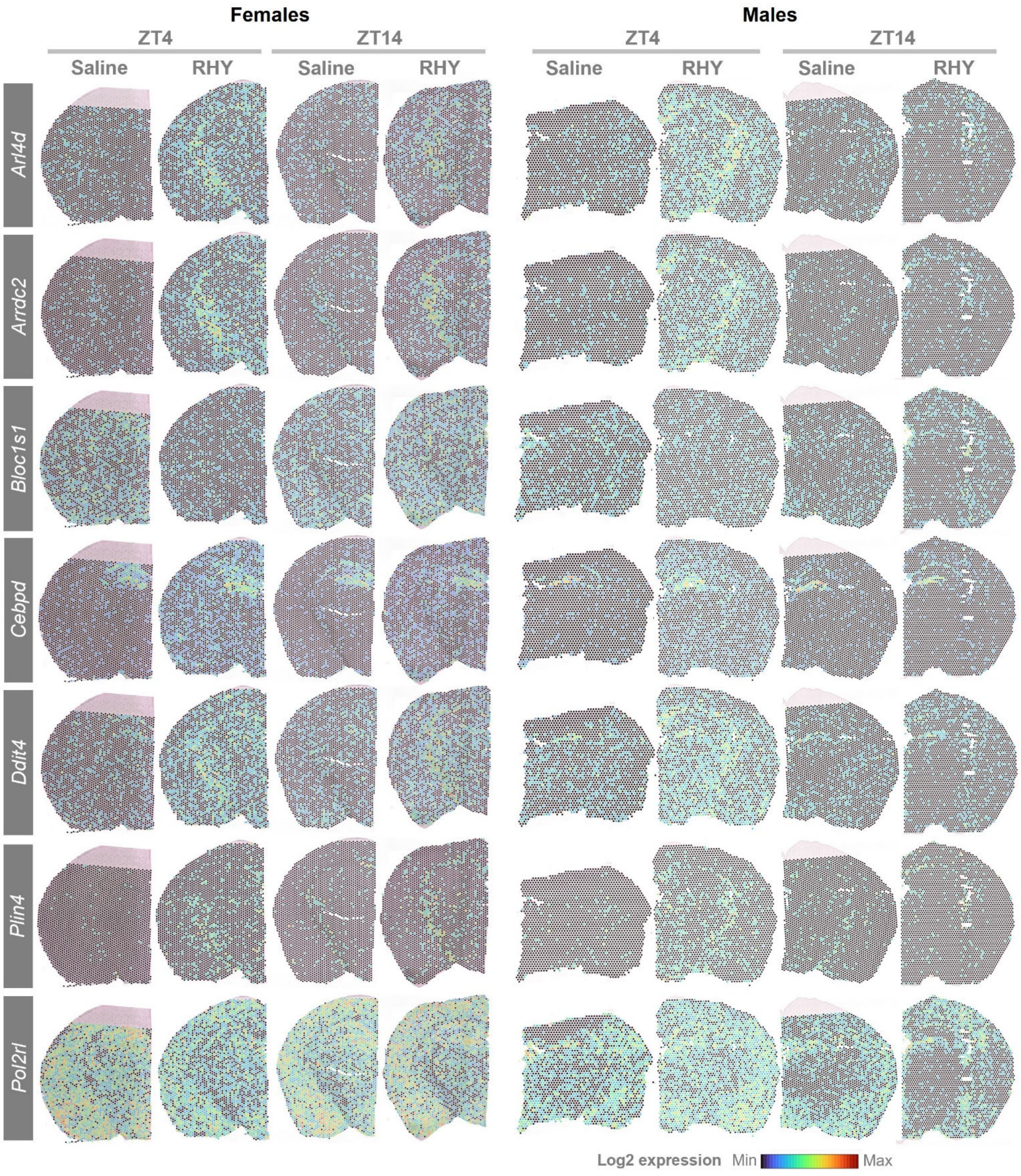

Figure S5. Spatial gene expression maps of selected DEGs found to be common between ZT4F, ZT4M, ZT14F and ZT14M. Color of spatial spots indicate Log2 gene expression under saline and RHY100 treatments for female and male mice. Values were normalized separately for males and female mice.

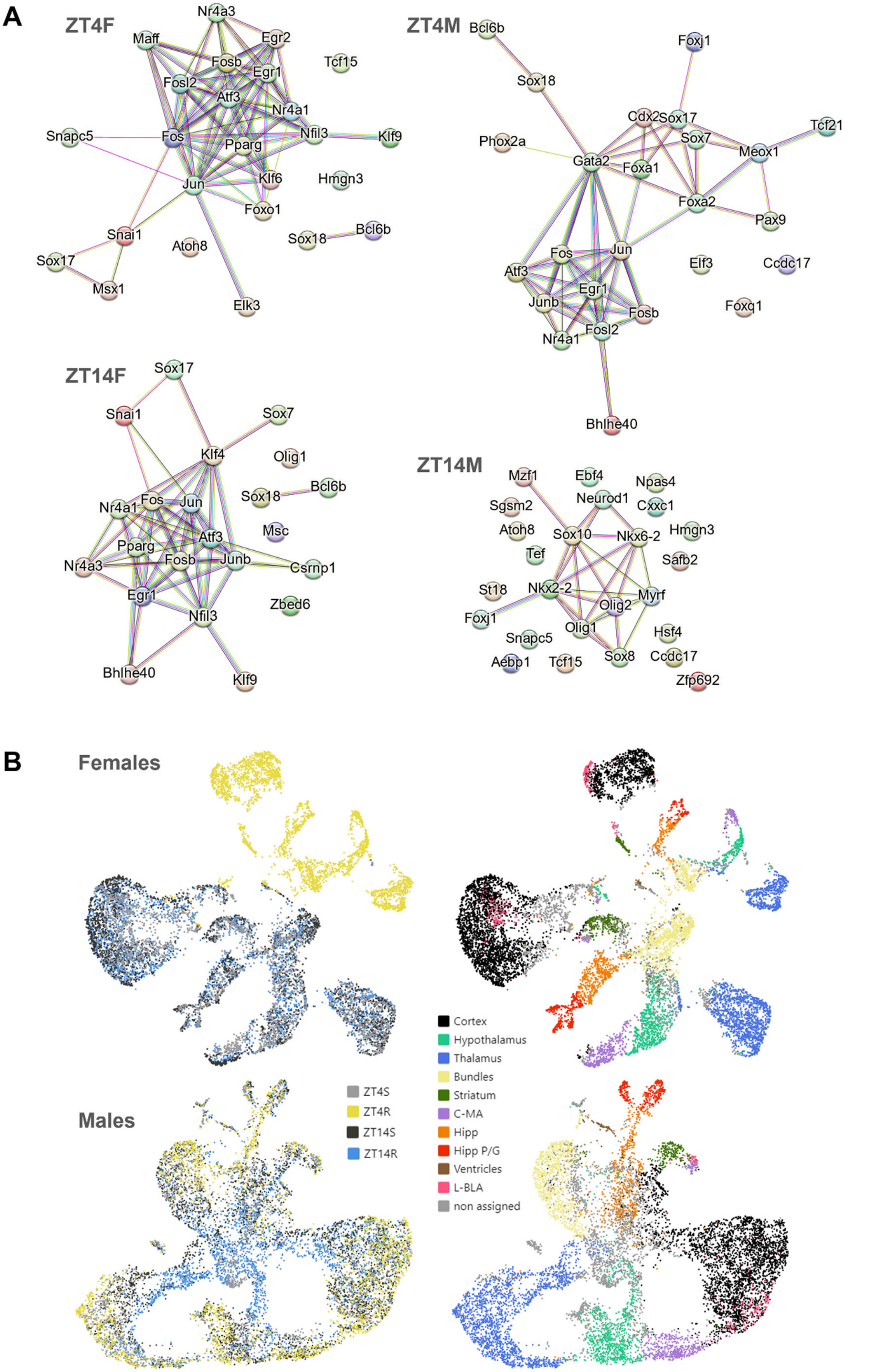
A
ZT4F
Nr4a3
Maff
Egr2
Fosb
Tcf15
Egr1
Fosl2
Atf3
Nr4a1
Snapc5
Fos
Nfil3
Klf9
Pparg
Klf6
Hmgn3
Jun
Foxo1
Sox18
Bcl6b
Snai1
Atoh8
Sox17
Msx1
Elk3
ZT4M
Bcl6b
Foxj1
Sox18
Cdx2
Sox17
Tcf21
Phox2a
Sox7
Meox1
Gata2
Foxa1
Foxa2
Pax9
Fos
Jun
Atf3
Elf3
Ccdc17
Egr1
Junb
Fosb
Foxq1
Fosl2
Nr4a1
Bhlhe40
ZT14F
Sox17
Snai1
Sox7
Klf4
Olig1
Sox18
Bcl6b
Nr4a1
Fos
Jun
Msc
Atf3
Pparg
Nr4a3
Fosb
Junb
Csrnp1
Egr1
Zbed6
Nfil3
Bhlhe40
Klf9
ZT14M
Mzf1
Ebf4
Npas4
Neurod1
Cxxc1
Sgsm2
Atoh8
Sox10
Nkx6-2
Hmgn3
Tef
Safb2
St18
Nkx2-2
Myrf
Foxj1
Olig2
Olig1
Snapc5
Hsf4
Aebp1
Tcf15
Sox8
Ccdc17
Zfp692
B
Females
Males
ZT4S
ZT4R
ZT14S
ZT14R
Cortex
Hypothalamus
Thalamus
Bundles
Striatum
C-MA
Hipp
Hipp P/G
Ventricles
L-BLA
non assigned

Figure S6. Transcription factors found to be enriched in ZT4F, ZT4M, ZT14F and ZT14M DEGs, and UMAP clustering of female and male spots related to brain regions.

(A) Interaction networks of the top 25 transcription factors found to be enriched using the CheA3 analysis in ZT4F, ZT4M, ZT14F and ZT14M DEGs. Pink lines denote experimentally determined; blue lines denote links reported from curated databases; black lines denote co-expression; yellow lines denote text-mining evidence; purple lines denote homology domains.

(B) Uniform manifold approximation and projection (UMAP) adequately represents the gene expression data of known brain regions. Top: UMAPs of the four female samples (ZT4S, ZT4R, ZT14S, ZT14R) identifying the samples (left) and brain regions (right). Bottom: same for the four male samples.

STAR★METHODS

RESOURCE AVAILABILITY

Lead Contact

Further information and requests for resources, protocols, and reagents should be directed to and will be fulfilled by the lead contact, Valérie Mongrain (valerie.mongrain@umontreal.ca).

Materials Availability

This study did not generate new unique reagents.

Data and code Availability

RNA-sequencing data have been deposited in the GEO database (https://www.ncbi.nlm.nih.gov/geo), and will be publicly available on the date of publication under accession numbers GSE217058 and GSE218537. Original Western blot images and quantifications are available as supplemental material (Table S1). The code generated to create the heatmaps, box plots and Venn diagrams is provided in supplemental material.

EXPERIMENTAL MODEL AND SUBJECT DETAILS

Animals

Male and female C57BL/6J were bred on site. Animals were housed in a 12 h light / 12 h dark cycle, at 24 ± 1°C, with water and food available *ad libitum*. All protocols were conducted in accordance with guidelines of the Canadian Council on Animal Care and approved by the *Comité d'éthique de l'expérimentation animale* of the *CIUSSS-NIM*.

METHOD DETAILS

EcoG and EMG electrode implantation

When mice reached 9-10 weeks-old, they were habituated to individual cages for two weeks. Subsequently, mice underwent implantation of electrodes for electrocorticography (EcoG) and electromyography (EMG) as detailed previously.[93,94] Female (n = 31 [n = 11 Saline, n = 9 RHY50, n = 11 RHY100], 12.7 ± 2.0 w old, 20.7 ± 1.7 g) and male (n = 29 [n = 9 Saline, n = 11 RHY50, n = 9 RHY100], 12.0 ± 3.2 w old, 26.3 ± 1.7 g) mice were implanted with electrodes under deep Ketamine/Xylazine anaesthesia (120/10 mg/kg, i.p. injection). Two gold-plated screws (diameter 1.1 mm) served as ECoG electrodes and were screwed through the skull over the right cerebral hemisphere (anterior 1.5 mm lateral to midline, 1.5 mm anterior to bregma; posterior 1.5 mm lateral to midline, 1 mm anterior to lambda). An additional screw placed on the right hemisphere (2.6 mm lateral to midline, 0.7 mm

posterior to bregma) was used as a reference. Three other screws were implanted over the left hemisphere as anchors. Two gold wires were inserted in neck muscles to serve as EMG electrodes. The ECoG and EMG electrodes were soldered to a connector and, together with the anchor screws, cemented to the skull. Once awake after anesthesia, mice received a subcutaneous injection of buprenorphine (0.1 mg/kg), and were allowed to recover for 5 days.

RHY preparation

It has been shown that 25-80 mg/kg of RHY modifies brain protein levels after one or several intraperitoneal injections.[62,63] Moreover, RHY has been detected in the mouse brain 10 min to 3 h after 50 mg/kg oral administration, and in the rat brain 15 min to 6 h after 10 mg/kg intravenous administration,[36] reaching maximum plasmatic concentrations 1-4 h after 37-50 mg/kg oral administration.[21,37] Therefore, two doses of RHY were tested and compared with vehicle (saline: NaCl 0.9 %): RHY 50 mg/kg (RHY50) and RHY 100 mg/kg (RHY100). RHY (Baoji Herbest Bio-Tech Co., Ltd, # 76-66-4) was diluted in NaCl 0.9 %, and homogenized the day before administration. RHY50 and RHY100 dilutions were kept at 4°C until use.

Protocols for ECoG/EMG recording

Following recovery from implantation surgery, mice were habituated to cabling conditions for one week before experiments. Their ECoG/EMG signals were then recorded during 48 h comprising 24 h of undisturbed/baseline (BL) conditions and 24 h under injection (INJ) conditions. On the INJ day, all mice received two intraperitoneal injections, one at ZT0 (i.e., light onset) and one at ZT11 (i.e., 1 h before light offset) of saline, RHY50 or RHY100. ECoG and EMG signals were amplified (Lamont amplifier) and sampled at 256 Hz with the software Harmonie (Natus, San Carlos, CA). Mice were sacrificed between ZT0 and ZT1 immediately after the INJ day (i.e., 24 h after the first and about 13 h after the second injection) by cervical dislocation, and brains were immediately dissected to sample the cerebral cortex, hippocampus and thalamus/hypothalamus. Brain tissues were quickly frozen on dry ice, and kept at -80°C until use.

Protein extraction and immunoblotting

Brain tissues were processed to extract total and synaptoneurosomal (SYN) proteins similar to previously performed.[93,95] Ice-cold modified RIPA buffer [10 mM HEPES, 2 mM EDTA, 1 mM EDTA, 0.5 mM DTT, protease and phosphatase inhibitors (Sigma-Aldrich)] was added to samples, which was followed by mechanical homogenization on ice (Pellet Pestle, Sigma Aldrich) until translucid (3 to 4 30-seconds trains). For total protein, a fraction of the homogenate was further sonicated (2 sec pulses, 5 sec pauses, 5 times) on ice, centrifuged at 13,000 rpm for 2 min at 4°C to remove cellular debris, and the supernatant was kept at -80°C for subsequent analysis. For SYN extraction, RIPA buffer was added to the remaining homogenate, vortexed, and centrifuged to remove nuclear/cell debris at 2,000 g and 4°C for 2 min. The supernatant was then passed through a 5 µm pore centrifugal filter (Ultrafree®-CL, Millipore), and centrifuged at 5,000 g and 4°C for 2 min. The filtrate was mixed and centrifuged at 5,000 g and 4°C for 15 min. The supernatants were then immediately resuspended in boiling RIPA buffer, and kept at -80°C until subsequent analysis.

For protein samples with concentration 0.6 µg/µL and above, 15 µg of protein were loaded on gels and separated by SDS-PAGE using an 8% acrylamide gel, and migration at 100 V for 65 min. Proteins were transferred

to a PVDF membrane (Bio-Rad) with transfer conditions of 100 V and 60 min. Membranes were blocked with blocking buffer (5% dry milk diluted in TBS [Tris-buffered saline]) for 1 h at room temperature. Primary antibodies were diluted in TBS-T blocking buffer (5% dry milk diluted in TBS [Tris-buffered saline 0.1% Tween 20]; except anti-phospho-EphA4 diluted in 5% BSA [bovine serum albumin] diluted in TBS-T), and were incubated overnight at 4°C. After washes with TBS-T, membranes were incubated with anti-Actin antibody (diluted in TBS-T blocking buffer) for 1 h at room temperature. After washing again, membranes were incubated for 1.5 h with secondary antibodies diluted in TBS-T blocking buffer for 1 h at room temperature. Membranes were revealed using Odyssey CLx imaging system (LI-COR).

Protocols for spatial transcriptomics

To study changes in the spatial transcriptome, saline and RHY100 treatments were compared. Four females and four males were used (12-13 w old): two females and two males received saline (one of each sex at ZT0 and one of each sex at ZT0 and ZT11), and two females and two males received RHY100 (one of each sex at ZT0 and one of each sex at ZT0 and ZT11). Mice were sacrificed at ZT4 or ZT14, and brains were immediately sampled, frozen on dry ice together with embedding in OCT compound (VWR International), and stored at -80°C until processing.

Tissue preparation for spatial transcriptomics

Tissue preparation was conducted according to the 10x Genomics Visium Spatial Gene Expression protocol (CG000240 Rev C). Ten µm coronal slices were cut at -23°C with a cryostat (HM525 NX Thermo Scientific or CM3050S Leica), and slices around 1.5 mm posterior to the bregma were mounted on chilled Visium Spatial Gene Expression slides (10x Genomics), and kept at -80°C for 2 to 4 days. Slides were then incubated in a thermocycler (using adaptor plate, 10x Genomics Accessory Kit, 1000194) for 1 min at 37°C, and immersed in Methanol for 30 min at -20°C. For Hematoxylin-Eosin staining (10X Genomics protocol CG000160 Rev A), slides were covered with isopropanol for 1 min at room temperature and, after air dry, covered with Hematoxylin for 7 min at room temperature. After washing, slides were covered with Bluing Buffer for 2 min at room temperature, washed, and covered with Eosin mix for 1 min at room temperature. Lastly, slides were dried using the thermocycler adaptor plate at 37°C for 5 min, and imaged using an Axio Imager M2 microscope (Zeiss, Canada).

Library preparation

Libraries were prepared according to 10x Genomics Visium Spatial Gene Expression protocol (CG000239 Rev D). Immediately after imaging, brain slices were covered with permeabilization enzyme and incubated on the thermocycler adaptor plate at 37°C for 6 min. After washing with 0.1X SSC, slices were covered with a reverse transcription master mix (including reverse transcription reagent, template switch oligonucleotides, reducing agent B and reverse transcription enzyme D), and incubated on the thermocycler adaptor plate at 53°C for 45 min. Then, the resulting cDNA (on slides) was incubated with 0.08M KOH for 5 min at room temperature, washed with EB buffer and incubated with second strand synthesis mix (including second strand reagent, primers and enzyme) for 15 min at 65°C. Slices were washed again with buffer EB, and denatured by incubation in 0.08M KOH for 10 min at room temperature. Solutions containing cDNA were transferred to a tube containing Tris 1 M pH 7.0 (1:8 final volume). Samples were mixed with cDNA amplification mix (containing amplification buffer and cDNA primers),

and processed in the thermal cycler (98°C for 3 min; 15 cycles of 15 sec at 98°C, 20 sec at 63°C and 1 min at 72°C; 1 min at 72°C). Then, cDNA was cleaned with SPRIselect beads (Beckman Coulter, Cat# B23318) 0.6X, washed with ethanol 80%, and resuspended with buffer EB. For cDNA fragmentation, end repair and A-tailing, samples were incubated in fragmentation mix (containing fragmentation buffer and enzyme) for 5 min at 32°C, and 30 min at 65°C. Samples were cleaned again with SPRIselect 0.6X and 0.8X, washed with ethanol 80%, and resuspended with buffer EB. Afterwards, samples were mixed with the adaptor ligation mix (containing ligation buffer, DNA ligase and adaptor oligos), and incubated at 20°C for 15 min. Post-ligation cleanup was done again with SPRIselect 0.8X, washed with ethanol 80%, and resuspended with buffer EB. Sample indexes i5 and i7, and amplification mix were added to the samples and processed in the thermal cycler: 45 sec at 98°C; 15 cycles of 98°C for 20 sec, 67°C for 30 sec, 72°C for 20 sec; and 72°C for 1 min. Then, cDNA was purified with SPRIselect 0.6X and 0.8X, washed with ethanol 80%, and resuspended with buffer EB. Libraries were stored at -20°C.

QUANTIFICATIONS AND STATISTICAL ANALYSES

Sleep scoring and analysis

ECoG and EMG signals were segmented into 4-s epochs, and the bipolar ECoG signal (signal difference between the anterior and posterior electrodes) and EMG were used to visually assign a vigilance state (wakefulness, SWS or PS) to each epoch by considering ECoG/EMG frequency and amplitude. Total time spent in each vigilance state, and the mean duration of individual bouts of vigilance states were averaged for the 12 h light and dark periods. The total number of bouts of 4, 8, 16, 32, 60, 120, 240, and 960 sec was calculated for the 24 h BL or INJ, for wake, SWS and PS separately. The proportion (percent) of time spent in each vigilance state and the mean duration of individual state bouts were also calculated for full 24 h. Hourly time-courses were calculated for mean time spent in each state and the total number of bouts.

For the ECoG activity analysis, two female mice were discarded and two male mice because of numerous artifacts in the ECoG signal (final analyzed sample: females n = 11 saline, 9 RHY50, 9 RHY100; males n = 8 saline, 10 RHY50, 9 RHY100). For analyzed mice, artifacts were excluded, and the bipolar ECoG signal was submitted to spectral analysis conducted using a Fast Fourier transform (FFT). ECoG activity during wake, SWS and PS was calculated for the full 24 h between 0.75 and 30 Hz with a 0.25 Hz resolution. Power spectra of the 24-h INJ recording were expressed as a percent of the mean power of all 0.25-Hz bins of all states during the 24-h BL for each mouse. The time course of SWS delta (1-4 Hz), delta 1 (0.75-2 Hz), delta 2 (2.5-4 Hz), theta (6-9 Hz), and sigma (10-13 Hz) activity, and of wake theta (6-9 Hz) and alpha (8-12 Hz) activity was calculated using averages per time interval as done previously.[94,96] In brief, to take into account the distribution of wakefulness and SWS sleep, SWS activity was average per interval for 12 equal intervals during light periods, and 6 equal intervals during dark periods; while wake activity was average for 6 equal intervals during light periods, and 12 equal intervals during dark periods. Then, relative activity was calculated for each interval as percent of the 24-h BL mean for each mouse.

Statistical analyses of sleep variables

Statistica 6.1 (StatSoft Inc./Tibco Software Inc., Palo Alto, CA, USA) was used to perform statistical analyses of sleep variables. Vigilance state variables calculated for the 24-h recordings and the 12-h light and dark periods were compared between treatments separately for female and male mice using one-way analyses of variance (ANOVAs). Vigilance state variables with significant treatment effects were further decomposed with post hoc Tukey tests. The percent time spent in each vigilance state was compared between treatments and sexes using two-way ANOVAs, and with significant treatment-by-sex interaction were decomposed with planned comparisons. Vigilance state variables calculated per hour or time interval as well as power spectra were analyzed using two-way repeated-measure ANOVAs (rANOVA), for which the significance level was adjusted using Greenhouse-Geisser or Huynh-Feldt correction. Significant treatment-by-time, treatment-by-intervals or recording day (BL vs. INJ)-by-frequency bin interactions were decomposed using planned comparisons or post hoc Tukey tests. Data are reported as mean and standard error of the mean (SEM), and the threshold for statistical significance was set to 0.05.

Protein level quantification and statistical analyses

Bands from immunoblots were analyzed using ImageJ (NIH).[97] Band intensity was quantified by calculating the area under the curve for the averaged pixel intensity along the vertical plane, which was normalized to actin, to a control sample (included on all membranes), and to the average of the total protein of the saline treatment. Values normalized to actin and control sample for phosphorylated and non phosphorylated forms of EPHA4 and GLUR1 were used to calculate the phosphorylation ratio, and were then normalized to the average ratio of the saline treatment. Prism 7 (GraphPad Software Inc., La Jolla, CA, USA) was used to perform statistical analyses and prepare figures. Protein levels were compared between treatments separately for female and male mice using one-way ANOVAs, and a significant treatment effect was decomposed using post hoc Tukey comparisons. Pearson correlations were computed between EPHA4, GLUR1, pGLUR1/GLUR1 and NR2B levels and sleep variables measured during time of higher effect of RHY (i.e., between ZT13 and ZT17 for time spent in SWS and PS, as well as SWS delta activity and wake alpha activity).

RNA sequencing

Paired-end dual indexed RNAseq was conducted using an Illumina NovaSeq6000 SP100 sequencer (Genome Quebec Innovation Centre, Montreal, Canada), at a sequencing depth of approximately 150M read pairs per sample (> 40 K reads per spot under tissue). RNAseq was performed according to instructions of 10x Genomics for the Visium Spatial Gene Expression kit: read 1, 28 cycles; i7 index read, 10 cycles; i5 index read, 10 cycles; read 2, 90 cycles.

RNAseq processing and gene expression analyses

Sequencing reads (demultiplexed FASTQ files) were aligned to the reference mouse genome (mm10) using the Space Ranger "spaceranger count" pipeline based on the splicing-aware aligner STAR. The pipeline initially trimmed the template switch oligo and poly-A sequences to improve the sensitivity of the alignment. The pipeline aligned reads to the genome, detected tissue spots by aligning the Hematoxylin-Eosin image using the fiducial frame of the capture area, and performed the barcode/unique molecular identifier counting. Reads mapped to the

transcriptome with high confidence were used for analysis. Then, the pipeline “spaceranger aggr” was used to find genes differentially expressed between ZT4S and ZT4R samples, and between ZT14S and ZT14R samples for females and males. Gene-spot matrices were analysed using Loupe Browser, and DEGs were considered significant when FDR < 0.05 (Benjamini-Hochberg correction for multiple comparisons).[98] Common DEGs between time point and sex were analyzed using VIB/UGent Bioinformatics & Evolutionary Genomics Venn diagram online calculator and R version 4.1.2. Clustered heatmap was created using the Ward.D2 clustering method. “Spaceranger aggr” was run again using ZT4S, ZT4R, ZT14S, and ZT14R from females, and afterwards from males, to obtain figures of spatial gene expression normalized per slide (thus, for males and females separately).

Functional gene ontology analyses

DEG lists were introduced in the DAVID annotation online tool, the Kyoto Encyclopedia of Genes and Genomes (KEGG) pathway annotation online tool, and the Ingenuity Pathway Analysis software (IPA, Qiagen) for functional analyses. Significant terms in the DAVID annotation online tool were considered when FDR < 0.05. In IPA, enrichment z-score was calculated considering transcripts Log2 fold change, and enriched terms for canonical pathway were considered when FDR ≤ 0.01; while enriched terms for predicted upstream elements were considered when FDR ≤ 0.0001, and z-score > 2; terms for predicted upstream transcription factors when FDR ≤ 0.01, and z-score > 1.5; terms for predicted upstream receptors when FDR ≤ 0.01, and z-score > 1.5; terms for predicted downstream functions when FDR ≤ 0.001. Enriched terms for biological processes (FDR < 0.0001), molecular function (FDR < 0.001), and KEGG (FDR < 0.001) were also reported. Transcription factor enrichment analysis was performed with the online tool ChIP-X Enrichment Analysis Version 3 (ChEA3),[47] and subsequent analysis of functional protein association networks was done with the online database STRING.[99]

ADDITIONAL RESOURCES

10x genomics: https://www.10xgenomics.com/products/spatial-gene-expression

VIB/UGent Bioinformatics & Evolutionary Genomics Venn diagram online calculator: https://bioinformatics.psb.ugent.be/webtools/Venn/

Ingenuity Pathway Analysis (IPA): https://digitalinsights.qiagen.com/products-overview/discovery-insights-portfolio/analysis-and-visualization/qiagen-ipa/

DAVID: https://david.ncifcrf.gov/

Kyoto Encyclopedia of Genes and Genomes (KEGG) pathway annotation online tool: https://www.genome.jp/kegg/

ChEA3: https://maayanlab.cloud/chea3/

STRING analysis: https://string-db.org/

CODES USED FOR TRANSCRIPTOMIC DATA ANALYSES

Code to align reads to genome

- Executing “spaceranger count”

```
spaceranger count \
--id=mysample \
--fastqs=/opt/bar/run1 \
--sample=sample1 \
--transcriptome=refdata-cellranger/GRCh38 \
--slide=V19L01-001 \
--image=highres.tff \
--area=A1
```

- Executing “spaceranger aggr”

library_id	molecule_h5	cloupe_file	spatial_folder
sample_id1	/path/to/outs/molecule_info.h5	/path/to/outs/cloupe.cloupe	/path/to/outs/spatial
sample_id2	/path/to/outs/molecule_info.h5	/path/to/outs/cloupe.cloupe	/path/to/outs/spatial

```
spaceranger aggr \
--id=mysample_aggr \ output directory
--csv=/opt/bar/aggr.csv \ aggregation CSV file
```

Code to visualize results

```
##Install packages
library(pheatmap)
library(xlsx)
library(RColorBrewer)
library(ggpubr)
library(ggplot2)
library(qusage)
library(ggplotify)
library(cowplot)
library(reshape2)
library(UpSetR)

## Choose colors for samples
sample_colors=c('darkblue','deepskyblue','deeppink4','deeppink1')
names(sample_colors)=c('ZT14M','ZT4M','ZT14F','ZT4F')

## Function to zoom specific modules/clusters
zoom.heatmap<-function(module,dat){
    dat=dat[module,]
    pheatmap(dat,clustering_method='ward.D',breaks=c(-5,-2,seq(-
1,1,by=.3),2,5),col=rev(brewer.pal(15,'RdYlBu')),fontsize_row=10, cluster_col=F)
}

## Function to package pathway analysis of modules in a heatmap
make.pathway_heatmap<-function(fish,ns_na=0.05,top=NA){
  first=T
  paths=c()
  for(n in names(fish)){
```

```
    sub = subset(fish[[n]],fdr<0.001)
    if(!is.na(top)){
      sub=head(sub,top)
    }
    paths = c(paths,as.character(rownames(sub)))
  }

  paths=unique(paths)
  first=T
  for(i in names(fish)){
    out = fish[[i]][paths,]
    out$pathway = rownames(out)
    out$cluster=i
    if(first){
      res=out
      first=FALSE
    }else{
      res=rbind(res,out)
    }
  }
  res$OR.odds.ratio  = as.numeric(as.character(res$OR.odds.ratio))
  d=reshape2::dcast(res,pathway~cluster,value.var='OR.odds.ratio')
  d2=reshape2::dcast(res,pathway~cluster,value.var='fdr')

  for(i in 2:ncol(d)){d[,i]=as.numeric(as.character(d[,i]))
  d[,i][!is.finite(d[,i])]=NA}
  rows=d[,1]
  d=d[,2:ncol(d)]
  d2=d2[,2:ncol(d2)]
  if(ns_na){
    d[d2>0.05]=NA
  }
  rownames(d)=rows

  ph=as.ggplot(pheatmap(log(1+d),fontsize_row=8,cluster_col=F,cluster_row=F,border_col='white'))
  return(ph)
}

## Function to calculate gene-set enrichment using fisher test
fisher_enrichment<-function(cluster_markers,pathway,universe,pathway_name){
enrichment_tables=list()
  for(cluster in 1:length(cluster_markers)){
    cluster_name=names(cluster_markers)[cluster]
    output <- lapply(pathway, function(x) {
    freq.table <- table(factor(universe %in% as.character(cluster_markers[[cluster]]),
                      levels = c(TRUE,FALSE)),
                        factor(universe %in% x,
                      levels = c(TRUE,FALSE)))

      fit <- fisher.test(freq.table, alternative = "greater")
      interSection <- intersect(cluster_markers[[cluster]], x)
      interSection <- paste(interSection, collapse = ",")
      return(value = c(NOM_pval = fit$p.value, INTERSECT = interSection,"OR"=fit$estimate))})

    term_names=character()
```

```
    for (pathway.term in 1:length(output)){
      term_names[pathway.term]=pathway[[pathway.term]][1]
    }

    results=data.frame(do.call("rbind",output))
    results$fdr=p.adjust(as.numeric(as.character(results$NOM_pval)),method = "BH")
    results=results[order(results$fdr),]
    enrichment_tables[[cluster_name]]=results
  }

  return(enrichment_tables)
}

## Load databases for gene-set enrichment
bp = readRDS('C5.BP.rds')
mf = readRDS('C5.MF.rds')
kegg = readRDS('C2.CP_KEGG.rds')

## Load excel files
files = list.files(pattern='xlsx')
files=files[c(1:4)]
dat = list()
for(f in files){dat[[f]]=read.xlsx(f,1)}

## Build list keeping genes that are DEGs (FDR<0.05) in at least one condition
genes = c()
degs=list()
for(n in names(dat)){
    s = subset(dat[[n]], dat[[n]][,5]<0.05)$FeatureName
    degs[[n]]=s
    genes = unique(c(genes,s))}

## Create Venn representation comparing the 4 sets of DEGs
venn.diagram(degs,'degs_across_conditions.dec2021.png')
up=upset(fromList(degs),order.by='freq')
up=upset(fromList(degs),sort.list(degs, decreasing=FALSE, method= c('ZT4F_ALLDEGs.xlsx',
'ZT4M_ALLDEGs.xlsx', 'ZT14F_ALLDEGs.xlsx', 'ZT14M_ALLDEGs.xlsx')))

## Create a table for the genes of interest. This table can be used as input for the pheatmap method
first=TRUE
for(n in names(dat)){
        dat[[n]]=subset(dat[[n]],!duplicated(FeatureName))
        sub  = subset(dat[[n]],FeatureName%in%genes)
        rownames(sub)=sub$FeatureName
        sub = sub[,c(4,5)]
        infos = unlist(strsplit(n,' '))[1]
        colnames(sub)=c(paste(infos,'log2FC',sep='.'),paste(infos,'FDR',sep='.'))
        if(first){
                first=F
                out  = sub
        }else{
                out=cbind(out,sub[rownames(out),])
        }
}
```

```
## Produce heatmap of the fold-changes. Scale by row to put emphasis on relative differences across conditions
out.fc = out[,grepl('log2FC',colnames(out))]
ph=pheatmap(na.omit(out.fc),clustering_method='ward.D2',breaks=c(-5,-2,seq(-1,1,by=.5),2,5),col=rev(brewer.pal(15,'RdYlBu')))

## Find clusters using the tree produced by pheatmap. The number of desired clusters can be changed with the k parameter
meta = data.frame(cutree(ph$tree_row,k=7))
meta[,1]=paste0('C',meta[,1])
colnames(meta)[1]='cluster'

gg_color_hue <- function(n) {
  hues = seq(15, 375, length = n + 1)
  hcl(h = hues, l = 65, c = 100)[1:n]
}
cols=gg_color_hue(7)
cols.list=list()
cols.list[['cluster']]=cols
names(cols.list[['cluster']])=paste0('C',1:7)
out.fc=out.fc[,c('ZT4F.log2FC','ZT4M.log2FC','ZT14F.log2FC','ZT14M.log2FC')]
## Redo heatmap by adding cluster annotation on the leaves of the tree
ph=as.ggplot(pheatmap(clustering_method='ward.D2',annotation_row=meta,na.omit(out.fc),breaks=c(-5,-2,seq(-1,1,by=.3),2,5),col=rev(brewer.pal(11,'RdYlBu')),cluster_col=F,fontsize_row=10,annotation_colors=cols.list))

## Create a temporary table to make plots by module
tmp = out.fc
tmp$module = meta[rownames(tmp),1]
tmp$gene = rownames(tmp)
m=melt(tmp)

m$variable = gsub('\\.log2FC','',m$variable)
m$variable = factor(m$variable,levels=c('ZT4F','ZT4M','ZT14F','ZT14M'))

## Distribution of the fold-changes across clusters
ag =aggregate(m$value,by=list(module=m$module,variable=m$variable),mean)
colnames(ag)[3]='value'

p3=ggplot(na.omit(m),aes(variable,value,fill=variable))+geom_line(aes(group=gene),alpha=.1,col='black')+geom_boxplot(outlier.shape=NA,size=.25)+facet_wrap(~module,ncol=3,scales='free_y')+geom_hline(yintercept=0,col='red',linetype='dashed')+theme_bw()+theme(axis.text.x=element_text(angle=90,hjust=1),strip.background=element_blank())+geom_point(alpha=.2,size=.1,position=position_jitter(width=.1),col='black')+scale_fill_manual(values=sample_colors)+ylab('log2FC')+xlab('')+geom_line(data=ag,aes(group=module,col=module),size=1,alpha=.8)+guides(col=F,fill=F)

## Bind heatmap and distribution of fold-changes
plot_grid(ph,p3,ncol=2,rel_widths=c(1,2))

modules=list()
for(c in unique(meta[,1])){
  modules[[c]]=rownames(subset(meta,meta[,1]==c))
  write.table(modules[[c]],paste0(c,'.genes.csv'),quote=F,row.names=F,col.names=F)
}

zoom.heatmap(modules[['C1']],out.fc)
```

```
## Calculate the geneset enrichments for the identified modules
fish.bp = fisher_enrichment(modules,bp,unique(dat[[1]][,2]))
fish.mf = fisher_enrichment(modules,mf,unique(dat[[1]][,2]))
fish.kegg = fisher_enrichment(modules,kegg,unique(dat[[1]][,2]))
#fish.tft = fisher_enrichment(modules,tft,unique(dat[[1]][,2]))

tmp=na.omit(tmp)

## Create correlation plots across module
a=ggplot(tmp,aes(ZT4F.log2FC,ZT4M.log2FC,col=module))+geom_point(size=.2)+stat_cor(col='black',size=2)+facet_wrap(~module,ncol=7)+geom_hline(yintercept=0,linetype='dashed')+geom_vline(xintercept=0,linetype='dashed')+theme_bw()+theme(strip.background=element_blank())+guides(col=F)
b=ggplot(tmp,aes(ZT14F.log2FC,x=ZT14M.log2FC,col=module))+geom_point(size=.2)+stat_cor(col='black',size=2)+facet_wrap(~module,ncol=7)+geom_hline(yintercept=0,linetype='dashed')+geom_vline(xintercept=0,linetype='dashed')+theme_bw()+theme(strip.background=element_blank())+guides(col=F)
c=ggplot(tmp,aes(ZT4F.log2FC,ZT14F.log2FC,col=module))+geom_point(size=.2)+stat_cor(col='black',size=2)+facet_wrap(~module,ncol=7)+geom_hline(yintercept=0,linetype='dashed')+geom_vline(xintercept=0,linetype='dashed')+theme_bw()+theme(strip.background=element_blank())+guides(col=F)
d=ggplot(tmp,aes(x=ZT14M.log2FC,ZT4M.log2FC,col=module))+geom_point(size=.2)+stat_cor(col='black',size=2)+facet_wrap(~module,ncol=7)+geom_hline(yintercept=0,linetype='dashed')+geom_vline(xintercept=0,linetype='dashed')+theme_bw()+theme(strip.background=element_blank())+guides(col=F)

## Bind plots
plot_grid(a,c,b,d,ncol=1)

## Create heatmaps for the gene-set enrichments results
make.pathway_heatmap(fish.bp)
make.pathway_heatmap(fish.mf)
make.pathway_heatmap(fish.kegg)

for(i in 1:length(fish.bp)){
fish.bp[[i]]$pathway=gsub('_',' ',gsub('GO_','',rownames(fish.bp[[i]])))
fish.bp[[i]]$OR.odds.ratio  = as.numeric(fish.bp[[i]]$OR.odds.ratio)}

plots=list()
for(i in 1:length(fish.bp)){
  top6 = head(fish.bp[[i]])
  top6$pathway=factor(top6$pathway,levels=top6[order(top6$OR.odds.ratio),'pathway'])
  plots[[i]]=ggplot(top6,aes(pathway,-log10(fdr)))+geom_col(fill=cols[i])+coord_flip()+theme_bw()}

# Find the 18 DEGs in common
in_common=Reduce(intersect,degs)

# In what module are they?
tmp[in_common,c('module','gene')]
```
